# An approach using *Caenorhabditis elegans* screening novel targets to suppress tumour cell proliferation

**DOI:** 10.1111/cpr.12832

**Published:** 2020-05-25

**Authors:** Yu‐Qin Mao, San‐Feng Han, Shi‐Long Zhang, Zheng‐Yan Zhang, Chao‐Yue Kong, Hui‐Ling Chen, Zhan‐Ming Li, Pei‐Ran Cai, Bing Han, Li‐Shun Wang

**Affiliations:** ^1^ Key Laboratory of Whole‐period Monitoring and Precise Intervention of Digestive Cancer (SMHC) Minhang Hospital Fudan University Shanghai China; ^2^ Institute of Fudan‐Minhang Academic Health System Minhang Hospital Fudan University Shanghai China

**Keywords:** *C elegans*, cell proliferation, target, tumour therapy

## Abstract

**Objectives:**

Tumour cell proliferation requires high metabolism to meet the bioenergetics and biosynthetic needs. Dauer in *Caenorhabditis elegans* is characterized by lower metabolism, and we established an approach with *C elegans* to find potential tumour therapy targets.

**Materials and methods:**

RNAi screening was used to find dauer‐related genes, and these genes were further analysed in *glp‐1(−)* mutants for tumour‐suppressing testing. The identified tumour‐related genes were verified in clinical tumour tissues.

**Results:**

The lifespan of *glp‐1(−)* mutants was found to be extended by classical dauer formation signalling. Then, 61 of 287 kinase‐coding genes in *Caenorhabditis elegans* were identified as dauer‐related genes, of which 27 were found to be homologous to human oncogenes. Furthermore, 12 dauer‐related genes were randomly selected for tumour‐suppressing test, and six genes significantly extended the lifespan of *glp‐1(−)* mutants. Of these six genes, *F47D12.9*, *W02B12.12* and *gcy‐21* were newly linked to dauer formation. These three new dauer‐related genes significantly suppressed tumour cell proliferation and thus extended the lifespan of *glp‐1(−)* mutants in a longevity‐ or dauer‐independent manner. The mRNA expression profiles indicated that these dauer‐related genes trigged similar low metabolism pattern in *glp‐1(−)* mutants. Notably, the expression of homolog gene DCAF4L2/*F47D12.9*, TSSK6/*W02B12.12* and NPR1/*gcy‐21* was found to be higher in glioma compared with adjacent normal tissue. In addition, the high expression of TSSK6/*W02B12.12* and NPR1/*gcy‐21* correlated with a worse survival in glioma patients.

**Conclusions:**

Dauer gene screening in combination with tumour‐suppressing test in *glp‐1(−)* mutants provided a useful approach to find potential targets for tumour therapy via suppressing tumour cell proliferation and rewiring tumour cell metabolism.

## 1 INTRODUCTION

Cancer remains one of the leading causes of death due to the late diagnosis, poor prognosis, metastasis and frequent occurrence of drug resistance.^1^ Usually, the treatment for tumour is surgery, chemotherapy, radiotherapy, complemented with immunotherapy and hormone therapy.^2^, ^3^ These various treatments for cancer may have not been able to fully meet the clinical needs, and they frequently bring a variety of side effects to patients.^4^, ^5^ Therefore, tumour still needs new treatment strategy.

The most fundamental trait of tumour cells involves their ability to sustain proliferation.^6^ In comparison with the normal tissues, high proliferating tumour cells need large amount of energy and biosynthetic precursors of macromolecules as building blocks for new cells.^7^ In clinical, patients who carry cancer cells with low proliferation can survive for relative long periods—in some cases indefinitely—without relapsing.^8^, ^9^ In addition, many of us may have in situ tumours, like breast, prostate and thyroid cancer, without recognizing it.^10^, ^11^ These findings suggest that these microscopic tumours are possibly dormant and need other proliferation signals to become lethal tumours.^10^

Evidence suggests that metabolism rewiring can suppress cancer incidence, delay tumour progression and inhibit metastasis.^12^, ^13^ As an example, calorie restriction impairs tumour cell proliferation, alters expression of cell cycle proteins, modifies tumour suppressor gene function and disrupts metabolic pathways.^14^ Therefore, tumour cell metabolism may be a potential target for tumour therapy. Dormancy is a special process with extremely low metabolism in nature.^15^ But there is no suitable model for screening dormancy‐related targets to suppress tumour cell proliferation.

By contrast to tumour cells, dauer, a dormancy in *C elegans*, which is characterized by their slow growth, maintains extremely low metabolic levels. The *C elegans* spontaneously enter dauer during the development of L2/L3 in response to adverse environmental conditions, such as high temperature, limiting amounts of food.^16^, ^17^, ^18^ Several signalling cascades including insulin‐like pathway, TGFβ‐like pathway, steroid hormone pathway and guanylyl cyclase pathway are documented to be critical in modulating nematode dauer formation.^16^, ^19^, ^20^, ^21^ In addition, *C elegans* is a fine model to study tumour. In *C elegans*, GLP‐1 signalling promotes a proliferative germ cell state and prevents germ cells from undergoing meiosis.^22^ Thus, loss of GLP‐1 signalling results in a severe proliferation defect and early meiotic entry, while constitutive activation yields a germ‐line tumour with all germ cells maintaining the germ cells in the stem cell state.^23^, ^24^ The expanding germ‐line tumour cells eventually perforate the gonad, invade throughout the worm body and lead to early animal death.^25^ Thus, tumour mutant in combination with dauer in *C elegans* may present a valuable model to find the targets suppressing tumour cell proliferation.

Here, we established an approach with *C elegans* for screening novel targets that rewire tumour cell metabolism and suppress proliferation. We uncovered dauer formation signals could rewire metabolism and extend the lifespan of *glp‐1(−)* mutants in a longevity‐ or dauer‐independent manner.

## 2 RESULTS

### 2.1 Classical dauer formation signals significantly extended the lifespan of *glp‐1(−)* mutants

**FIGURE 1:**
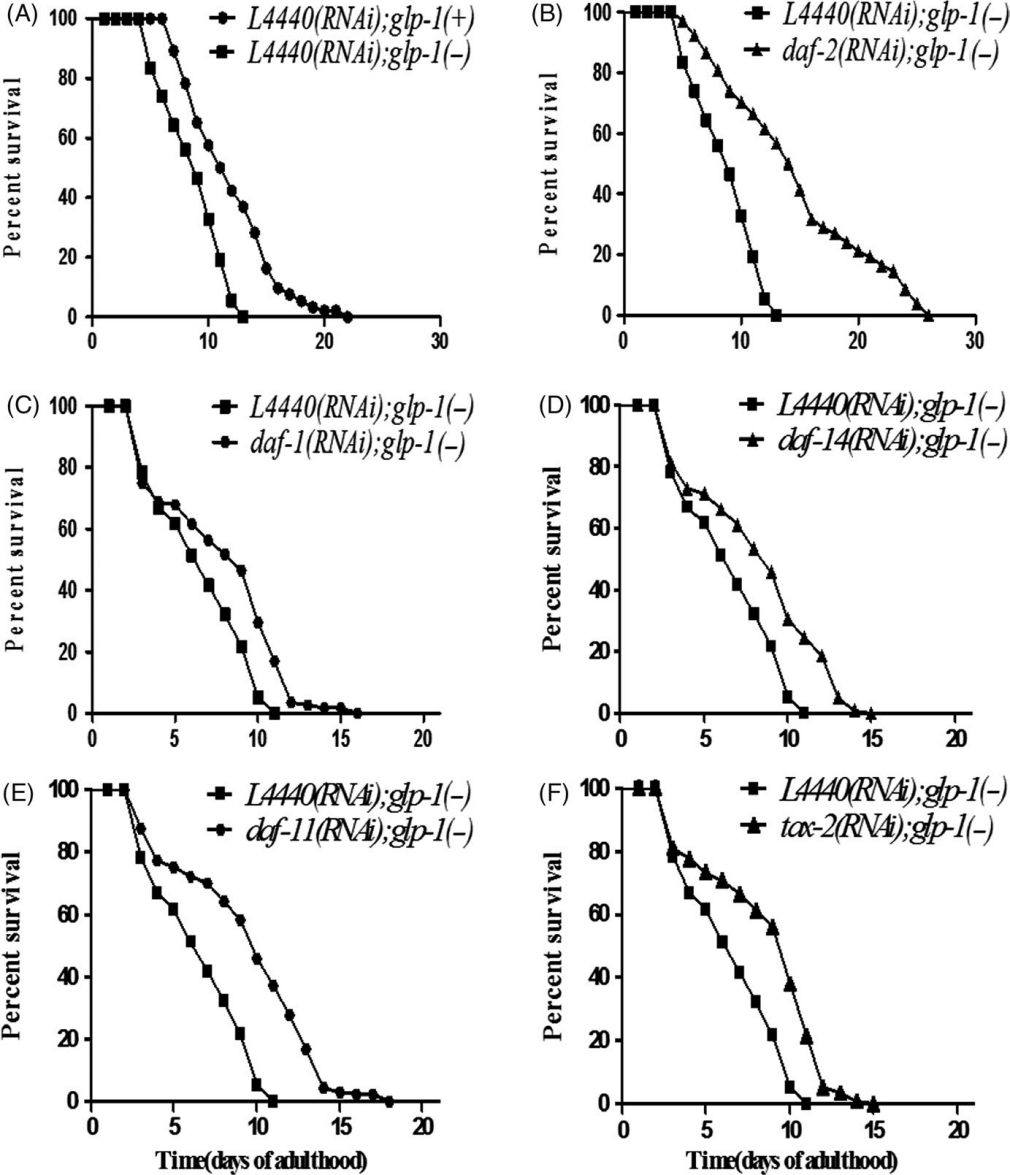
Classical dauer formation signals extend the lifespan of *glp‐1(−)* mutants. For each chart, experimental and control animals were grown in parallel. In this and other figures, *glp‐1(−)* refers to the null allele *glp‐1*(ar202), *glp‐1(+)* refers to N2 worms, and all RNAi‐treated animals are labelled accordingly. A, Lifespan curves of *glp‐1(+)* worms and *glp‐1(−)* mutants. B‐F, The lifespan curves of *daf‐*2 RNAi (B), *daf‐1* RNAi (C), *daf‐14* RNAi (D), *daf‐11* RNAi (E) and *tax‐2* RNAi (F)

It was reported that in *glp‐1(−)* mutants, the germ cells in the early stage of oogenesis could re‐enter into the mitotic cell cycle and overproliferate, making them resemble the overproliferation of tumour cell and shortening the lifespan of patients.^22^, ^23^ In this study, we tested whether the short lifespan in *glp‐1(−)*mutants could be extended by the classical dauer formation signals, which inhibit insulin‐like pathway,^21^ guanylyl cyclase pathway,^26^, ^27^, ^28^ TGFβ‐like pathway^29^ and steroid hormone pathway, respectively.^30^ As shown in Figure 1A, we found *glp‐1(−)* mutants suffered a shorter lifespan compared with N2 (*glp‐1(+)*) worms. Next, we knock down *daf‐2*, *daf‐1*, *daf‐14*, *daf‐11* and *tax‐2* by RNAi, respectively, which led to the activation of dauer formation pathway. Compared to *L4440* RNAi in *glp‐1(−)* mutants, these dauer formation signals extended the mean survival rate of *glp‐1(−)* mutants by 114.28%, 28.57%, 28.57%, 42.86% and 42.86%, respectively, for *daf‐2*, *daf‐1*, *daf‐14*, *daf‐11* and *tax‐2* (Figure 1B‐F, Table S1), and all the *P* values for each dauer formation signal in three independent trials were <.001 (Table S1), indicating a solid contribution of dauer formation signals to the lifespan extension in *glp‐1(−)* mutants.

### 2.2 Classical dauer formation signals suppressed germ cell proliferation

**FIGURE 2:**
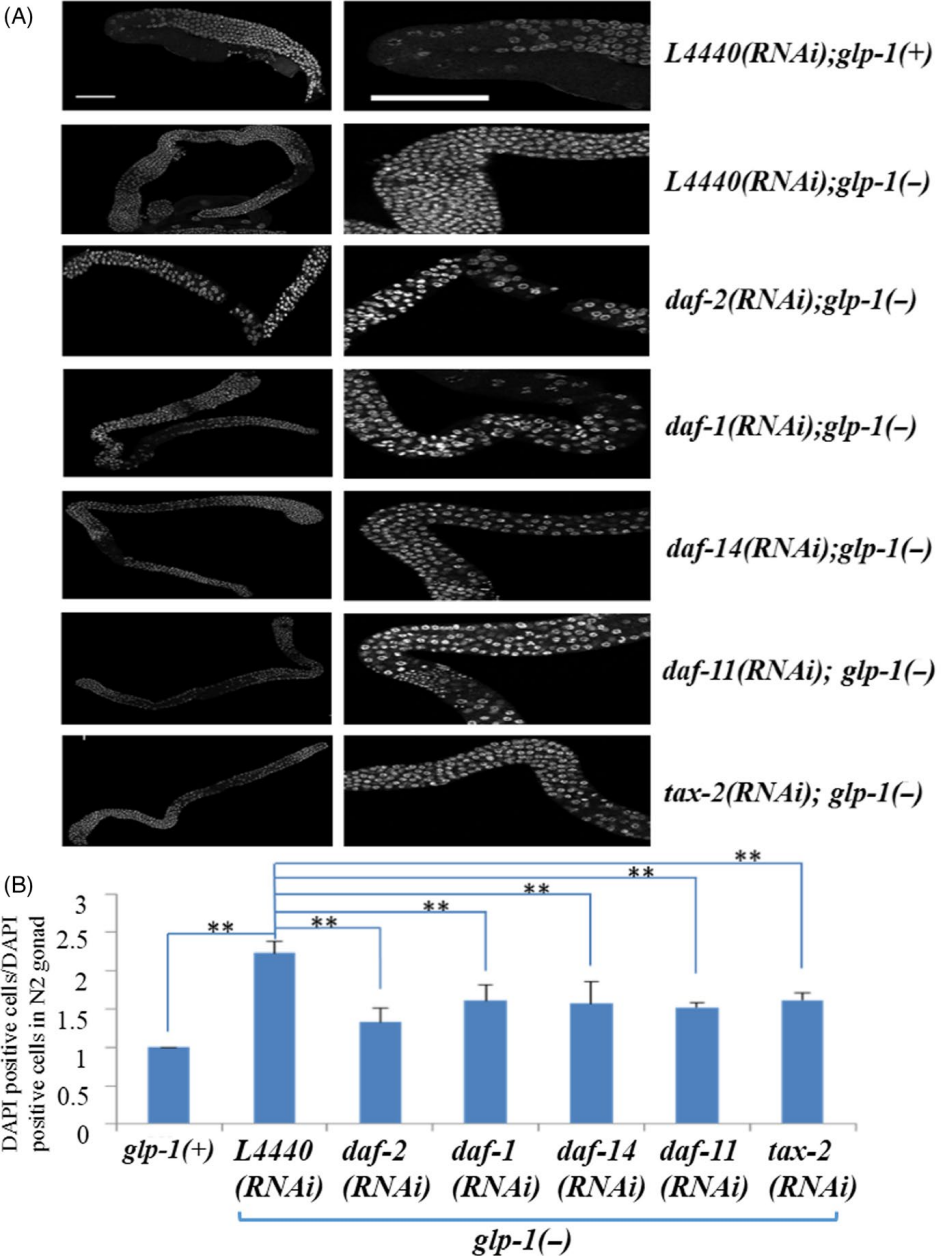
Classical dauer formation signals suppress germ cell proliferation in *glp‐1(−)* mutants. Adult animals were stained with the DNA‐intercalating dye DAPI. Left panels, the whole gonad. Right panels, midpoints of the gonad arms. A, *glp‐1(−)* mutants lack oocytes and have many undifferentiated germ cells in their gonads. Knockdown of *daf‐2*, *daf‐1*, *daf‐14*, *daf‐11* and *tax‐2* by RNAi, respectively, they have far fewer undifferentiated germ cells and maintain the integrity of their gonads. Representative of n = 3 independent experiments. B, Knockdown of classical dauer formation signals reduced undifferentiated germ cells was detected and analysed using one‐way ANOVA test followed by Bonferroni correction for post hoc test (*P < .05,**P < .01 for *t* test); data shown are mean ± SD

Next, we asked whether these classical dauer formation signals extend the lifespan via suppressing the germ cell excessive proliferation in *glp‐1(−)* mutants. We knocked down these dauer formation signals in these worms, then dissected the entire gonads at day 4 of adulthood and detected the germ cell number with DNA‐intercalating dye DAPI. Our results showed that *glp‐1(−)* mutants had more undifferentiated germ cells than *glp‐1(+)* worms (Figure 2A,B). Furthermore, knockdown of *daf‐2*, *daf‐1*, *daf‐14*, *daf‐11* and *tax‐2* resulted in reduced undifferentiated germ cells compared with the counterparts feeding with *L4440* in *glp‐1(−)* mutants (Figure 2A,B, Table S2), and all the *P* values were less than .001. Collectively, these data suggested that activation of classical dauer formation signals suppressed tumour cell proliferation.

### 2.3 Three novel dauer‐related genes extended the lifespan of *glp‐1(−)* mutants

Most of the homologous of classic dauer signals in human had antitumour effect, as shown in Table 1. Hence, we used the *glp‐1(−)* mutants to screen novel genes capable of suppressing tumour cell proliferation. Protein kinases play an indispensable role in regulating cell proliferation and function, which makes them perfect targets for screening tumour‐related genes.^31^, ^32^, ^33^

**TABLE 1:** The homolog genes of dauer formation genes in clinical

Dauer genes	Homologous genes	Clinical application
*daf‐2*	IGF‐1	Chronic lymphocytic leukaemia,^74^ metastatic Ewing family tumours,^75^ pancreatic cancer^76^
*daf‐1*	ACVR1C	Retinoblastoma^77^
*akt‐2*	AKT	Multiple myeloma, gastric cancer, ovarian^78^
*tax‐2*	CNGB3	Peritoneal endometriosis^79^
*let‐23*	EGFR	Colorectal cancer^52^, ^80^
*aak‐1*	PRKAA1	Chronic myelomonocytic leukaemia^81^

**FIGURE 3:**
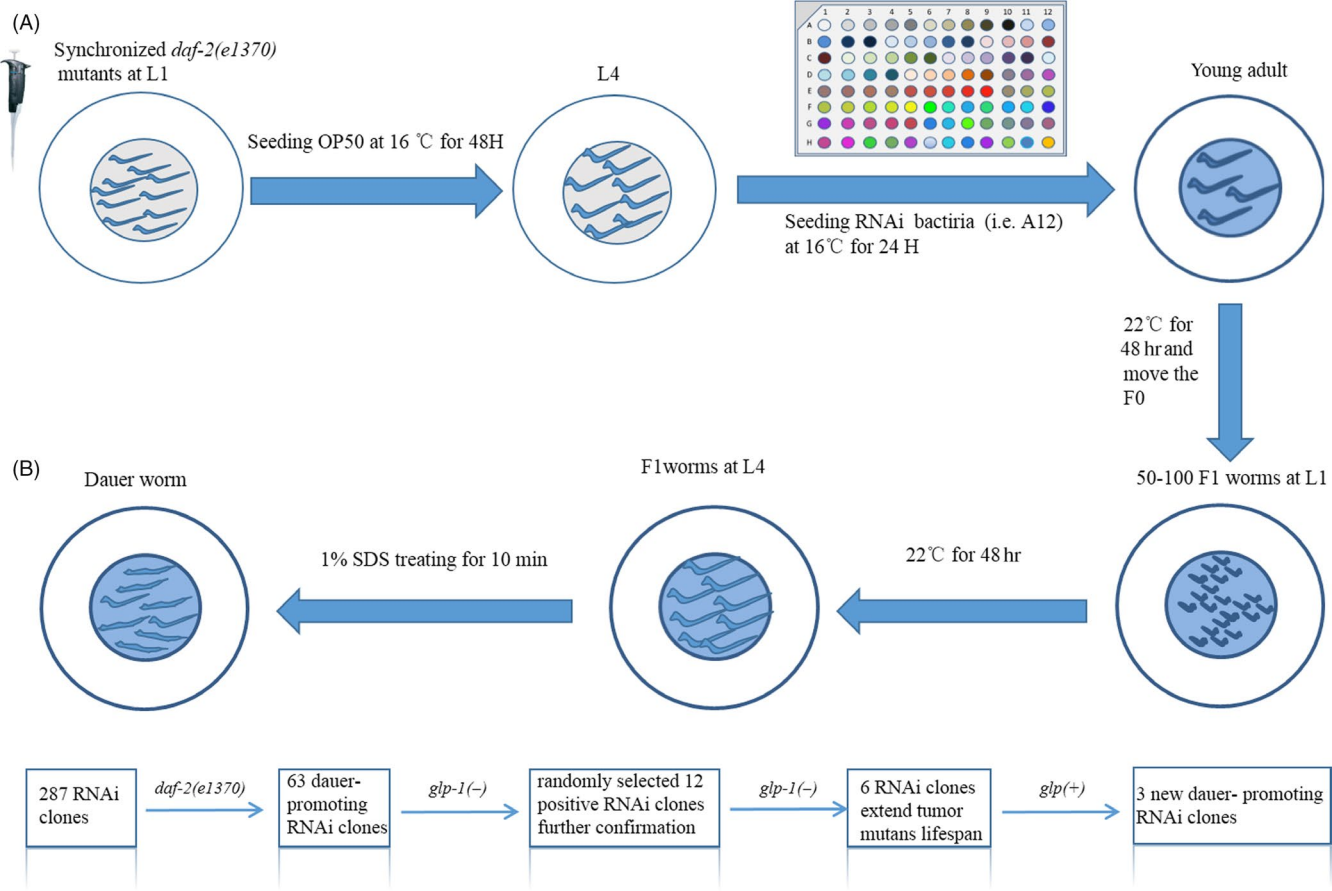
Experimental design of genetic screen. A, Schematic of the procedures for the primary screen using *daf‐2(e1370)* mutants, which can spontaneously enter the stage of dauer larva under certain temperature. Various colours represent different RNAi clones from the single‐gene knockout RNAi library. B, The working flow of screens and subsequent retests

**FIGURE 4:**
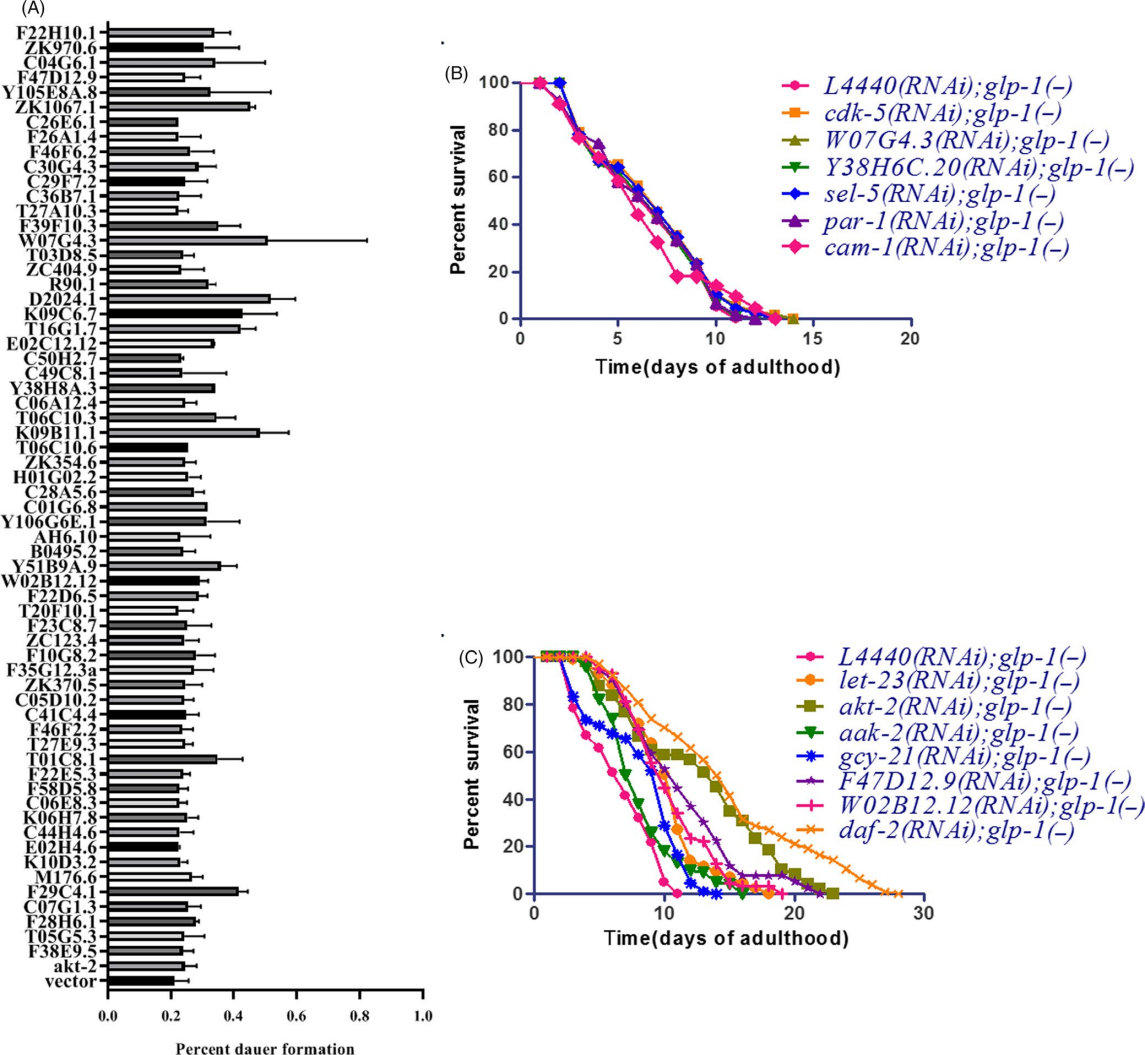
Kinases promote dauer formation and extend the lifespan of *glp‐1(−)* mutants by RNAi. A, A genomewide screen of the *Escherichia coli* HT115 single‐gene knockout library identified 61 genes that promote dauer formation by >20% (*P* < .05, log‐rank test). The dauer‐promoting genes presented in *daf‐2(e1370)* mutants at 22°C. Randomly selected 12 genes were knocked down (B, C) in *glp‐1(−)* mutants, and the survival per cent was detected. Six of these genes did not extend the lifespan of *glp‐1(−)* mutants (B), while the remaining six genes extended the lifespan of the *glp‐1(−)* mutants (C)

**FIGURE 5:**
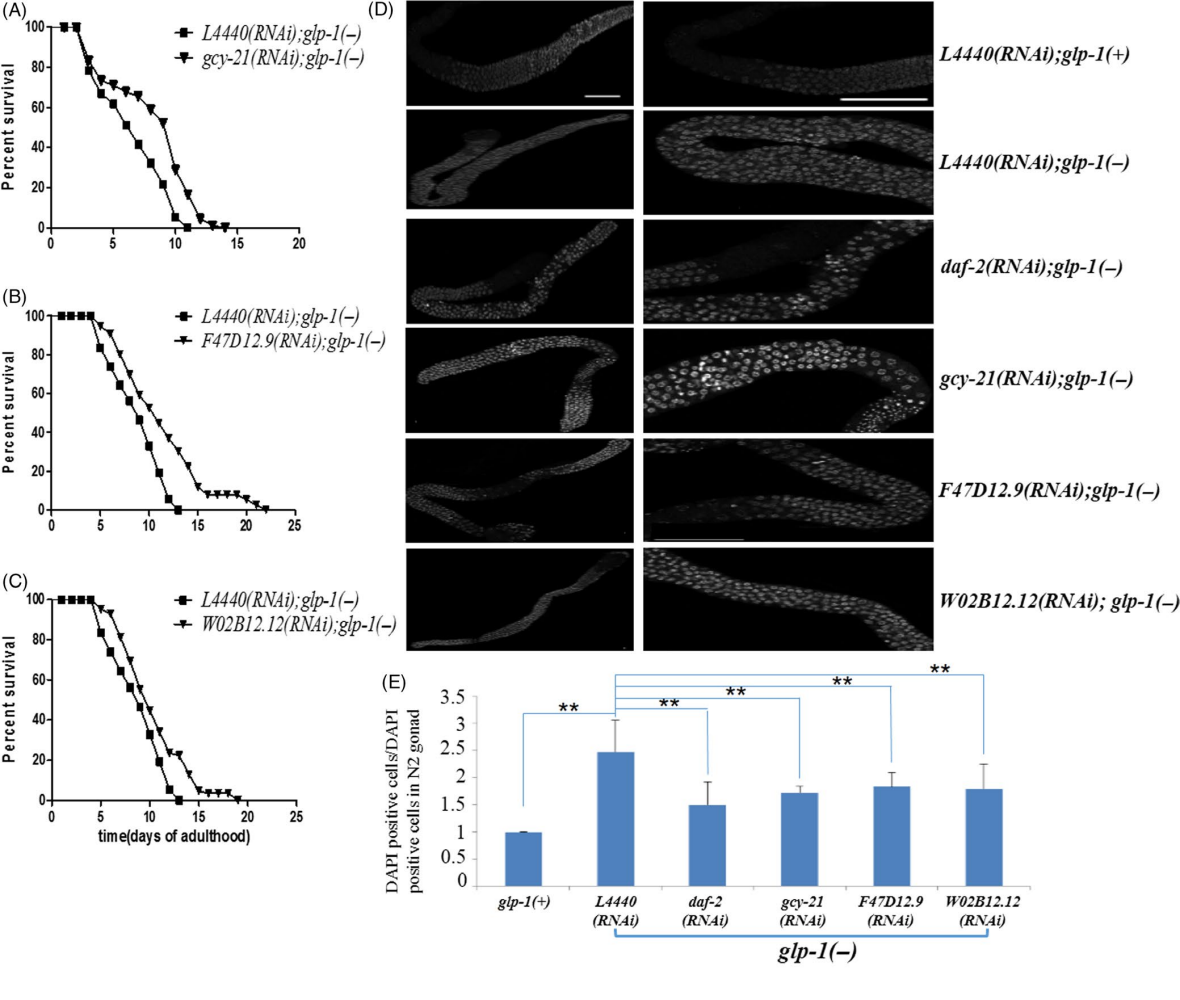
Dauer‐related genes extend the lifespan of *glp‐1(−)* mutants and reduce germ cell number in *glp‐1(−)* mutants. (A‐C) The lifespan curves of *gcy‐21* RNAi (A), *F47D12.9* RNAi (B), *W02B12.12* RNAi in *glp‐1(−)* mutants. Adult animals were stained with the DNA‐intercalating dye DAPI. Left panels, the whole gonad. Right panels, midpoints of the gonad arms. (D) *glp‐1(−)* mutants lack oocytes and have many undifferentiated germ cells in their gonads. Knockdown of *gcy‐21*, *F47D12.9* and *W02B12.12*, they have far fewer undifferentiated germ cells and maintain the integrity of their gonads. Representative of n = 3 independent experiments. E, Knockdown of *gcy‐21*, *F47D12.9* and *W02B12.12* reduced undifferentiated germ cells was detected and analysed using one‐way ANOVA test followed by Bonferroni correction for post hoc test (*P < .05,**P < .01 for *t* test); data shown are mean ± SD

Worms encode about 438 kinase‐coding genes.^34^, ^35^ To screen these genes, an RNAi library contains 287 kinase‐coding genes were established (Figure 3A). We optimized conditions for RNAi library screening and performed the screening (see Figure S1). Sixty‐one genes were identified to promote dauer formation after RNAi (Figures 3B, 4A), of which 27 human homologs genes were well‐studied oncogenes (Table 2), demonstrating the potential value of this screening approach. Then, we randomly selected 12 genes for further evaluation. As a result, knockdown of *cam‐1, par‐1, cdk‐5*, *W07G4.3*, *Y38H6C.20* and *sel‐5* showed no effect on lifespan extension in *glp‐1(−)* mutants (Figures 3B, 4B), while knockdown of genes including *aak‐2, akt‐2*, *let‐23*, *F47D12.9*, *W02B12.12* and *gcy‐21* extended the lifespan of *glp‐1(−)* mutants (Figures 3B, 4C). Interestingly, *F47D12.9*, *W02B12.12* and *gcy‐21* were first identified to be involved in dauer formation. To confirm our finding, we knock down these three genes, respectively, as a consequence, the survival of *glp‐1(−)* mutants was significantly increased, and all the *P* values were <.001(Figure 5A‐C, Table S3). Furthermore, at day 4 of adulthood, these worms had less germ cell than *glp‐1(−)* mutants feeding with *L4440*, (Figure 5D, Table S4), this was associated with the decreased cell proliferation (Figure 5E), and all the *P* values were <.001.

**TABLE 2:** The homologous genes of dauer‐related genes in regulating tumour cell proliferation and metabolism

Dauer‐related genes	Homologous genes	Related research in Tumour
*akt‐2*	AKT2	PTEN‐deficient tumours^82^
*C06E8.3*	PIM3	Pancreatic cancer^83^
*C01G6.8*	ROR1	Chronic lymphocytic leukaemia ^84^
*F38E9.5*	TWF1	Lung adenocarcinoma ^85^
*T05G5.3*	CDK1	Melanoma ^86^
*C07G1.3*	CDK16	Hepatocellular carcinoma ^87^
*F29C4.1*	ACVR1C	Retinoblastoma^77^
*M176.6*	AXL	Melanoma^88^
*C44H4.6*	GSK3B	Pancreatic cancer^89^
*F22E5.3*	NPR1	Breast cancer^90^
*T01C8.1/aak‐1*	PRKAA1	Gastric cancer^91^
*T27E9.3*	CDK‐5	Glioma^92^
*F46F2.2*	CSNK1D	Breast cancer^93^
*C05D10.2*	MAPK15	Chronic myeloid leukaemia^94^
*ZK370.5*	PDK1	Breast cancer^95^
*ZC123.4*	CDK14	Oesophageal cancer^96^
*B0495.2*	CDK11B	Cholangiocarcinoma^97^
*H01G02.2*	CDK20	Hepatocellular carcinoma^98^
*K09B11.1*	Irak4	Chronic lymphocytic leukaemia^99^
*T06C10.3*	FER	Melanoma^100^
*R90.1*	TTBK2	Glioma^101^
*ZC404.9*	MAP4K3	Lung cancer^102^
*F46F6.2*	PKN3	Prostate cancer^103^
*ZK1067.1/let‐23*	ERBB4	Gastric cancer^104^
*Y105E8A.8*	WRAP53	Colorectal cancer ^105^
*F47D12.9*	DCAF4L2	Colorectal cancer^56^
*C04G6.1*	MAPK7	Osteosarcoma^106^

### 2.4 The lifespan extension of dauer‐related genes in *glp‐1(−)* mutants was independent of longevity and dauer period

**FIGURE 6:**
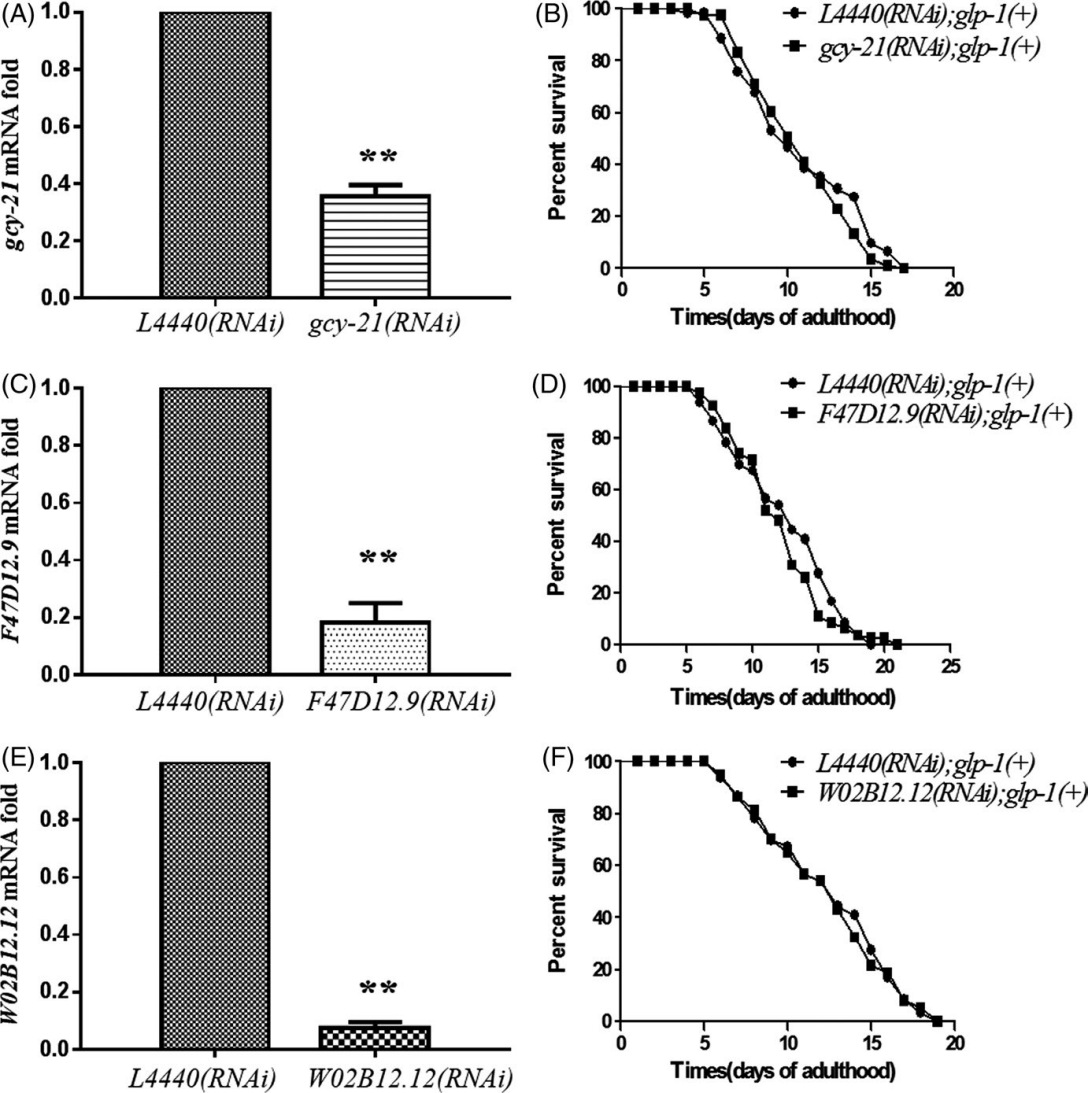
Dauer‐related genes extend the lifespan of *glp‐1(−)* mutants not N2 worms. A, The mRNA levels of *gcy‐21* in *glp‐1(+)* mutants feeding with *L4440* or *gcy‐21*. B, The survival curves of *gcy‐21* RNAi in the background of *glp‐1(+)* mutants. C, The mRNA of *F47D12.9* in *glp‐1(+)* mutants feeding with *L4440* or *F47D12.9*. D, The survival curves of *F47D12.9* RNAi in the background of *glp‐1(+)* mutants. E, The mRNA of *W02B12.12* in *glp‐1(+)* mutants feeding with *L4440* or *W02B12.12*. F, The survival curves of *W02B12.12* RNAi in the background of *glp‐1(+)* mutants. *Glp‐1(+)* refers to N2 worms. RNAi represents RNA interference. Forty cycles used for semi‐quantitative RT–PCR. Bars, SD. (*P < .05, **P < .01 for *t* test), data represent n = 3 independent experiments

Most of classical dauer formation signals were reported to extend the lifespan of *glp‐1(+)* worms; Kenyon et al^36^ had demonstrated that longevity signals could inhibit tumour growth. The *glp‐1(−)* mutants in our study were in the background of N2 (*glp‐1(+)*) worms. To test whether these three dauer‐related genes have inherent longevity effect, we knockdown these genes in *glp‐1(+)* worms, respectively, and no longevity effect was observed (Figure 6A‐F, Table S5), which suggested the lifespan extension in *glp‐1(−)* mutants was independent of longevity.

**FIGURE 7:**
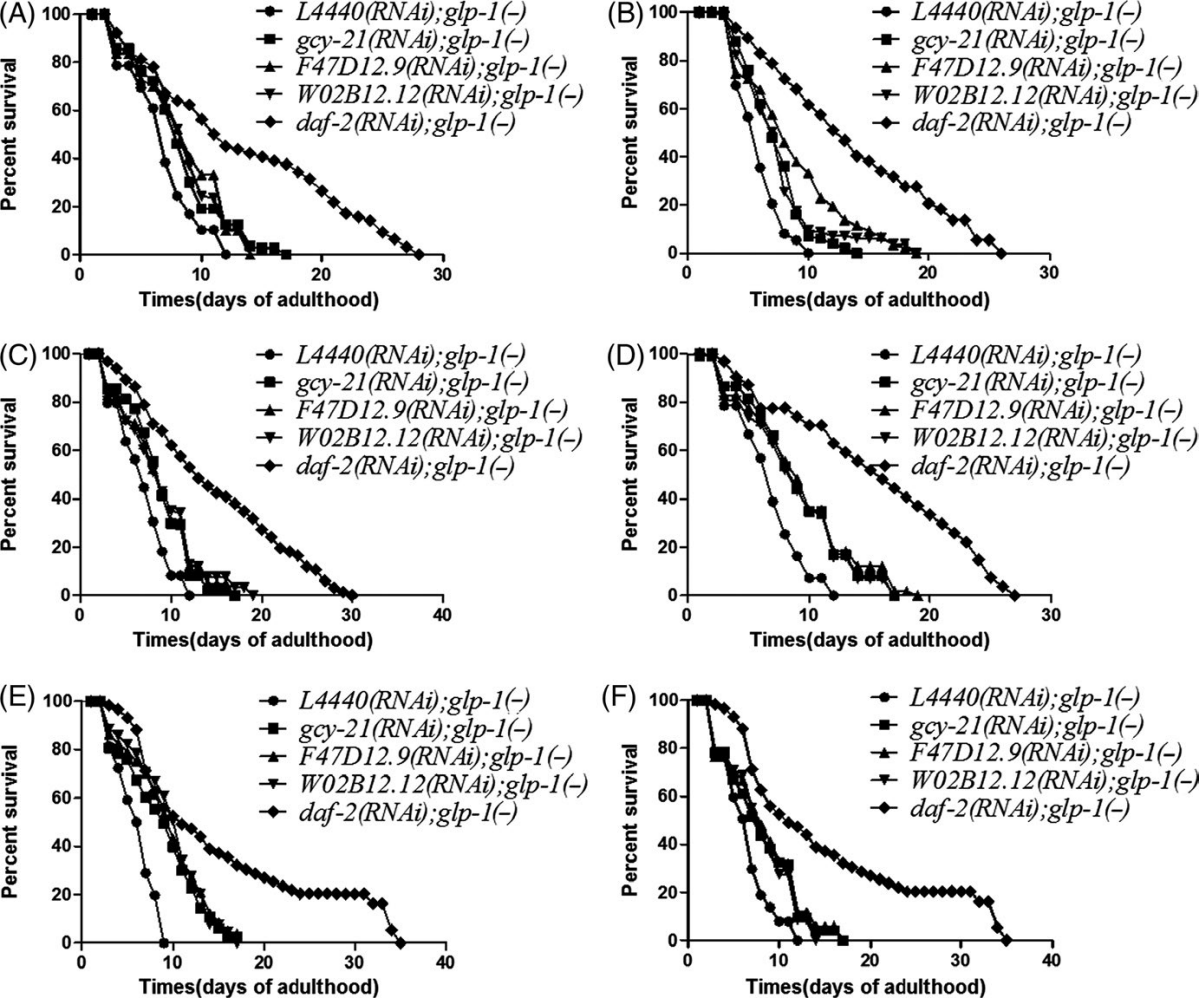
Dauer‐related genes extend the lifespan of *glp‐1(−)* independent of dauer stage. We investigated the effect of *daf‐2* RNAi, *gcy‐21* RNAi, *F47D12.9* RNAi or *W02B12.12* RNAi, respectively, at different developmental stages in *glp‐1(−)* mutants. A‐E, The survival rate for *daf‐2* RNAi, *gcy‐21* RNAi, *F47D12.9* RNAi or *W02B12.12* RNAi at (A) L1, (B) L2, (C) L3, (D) L4, (E) day 1 of adulthood and (F) day 4 of adulthood, respectively; they all extended the lifespan of *glp‐1(−)* mutants

The dauer occurs in the L2/L3 larval stage.^37^, ^38^, ^39^ To determine whether the dauer period contributes to lifespan extension in *glp‐1(−)* mutants, we knock down *daf‐2*, *gcy‐21*, *F47D12.9* and *W02B12.12* in *glp‐1(−)* mutants at different development stages including L1, L2, L3, L4, day 1 and day 4 of adulthood (Figure 7), We found the knockdown of these genes extended the lifespan of *glp‐1(−)* mutants at all developmental stages, and all the *P* values for each dauer‐related gene were <.001 (Figure 7A‐E, Table S6). These data showed that these dauer‐related genes extend the lifespan of *glp‐1(−)* mutants independent of dauer stage.

### 2.5 Dauer‐related genes rewired metabolism pathways in the *glp‐1(−)* mutants

To investigate the molecular mechanisms underlying the antitumour effect of dauer‐related genes, we compared their transcriptome profiles in worms. As shown in Figure S2A, compared to *glp‐1(+)* worms*,* the *glp‐1(−)* mutants had 5364 differentially expressed genes, of which 2514 genes were downregulated and 2850 genes were upregulated. In the *glp‐1(−)* mutants, 86, 32, 102, 38 downregulated genes and 1022, 36, 102, 84 upregulated genes were identified after the knockdown of *daf‐2*, *gcy‐21*, *F47D12.9* and *W02B12.12*, respectively (Figure S2B‐E).

Next, we analysed the differentially expressed genes. RNA‐related metabolism and processing were found to be significantly increased *glp‐1(−)* mutants when compared to *glp‐1(+)* worms evidenced by Gene Ontology analysis, while this increment was reduced in *daf‐2* RNAi groups (Figures S3 and S4). As shown in Figures S3 and S5, oxidoreductase activity was upregulated in *glp‐1(−)* mutants and it was reduced in *gcy‐21* RNAi group. *Glp‐1(−)* mutants increased the structural molecule activity and transferase activity, while the knockdown of *F47D12.9* or *W02B12.1*2 reversed them (Figures S3, S6 and S7).

**FIGURE 8:**
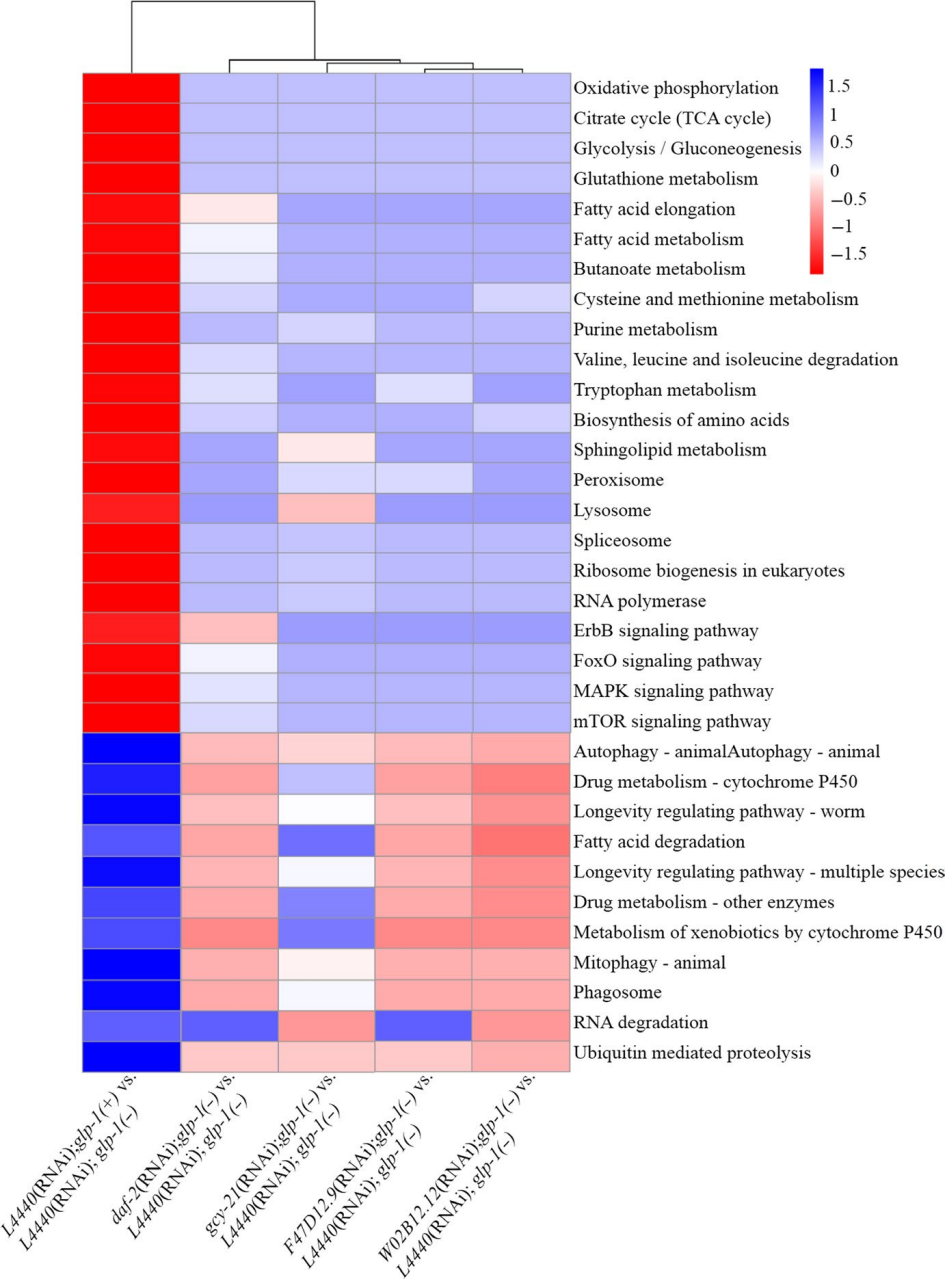
Dauer‐related genes rewired metabolism in *glp‐1(−)* mutants. A, KEGG pathway enrichment analysis of the identified differential expressed genes. Heatmap represents the differential expressed genes enriched in related pathway. The red colour represents the upregulated genes, and the blue colour represents the downregulated genes. The deeper the colour, the more genes enrich in related genes. Heatmap using enriched genes set with options: *z*‐score based row and column normalization

Furthermore, to better understand how dauer‐related genes affect tumour, Kyoto Encyclopedia of Genes and Genomes (KEGG) pathway enrichment analysis was performed. As shown in Figure 8, compared to *glp‐1(+)* worms, *glp‐1(−)* mutants upregulated glycolysis, amino acid metabolism, fat acid elongation, RNA polymerase, ribosome biogenesis, peroxisome and lysosome. However, these upregulated cellular metabolisms were reversed by the RNAi of *daf‐2*, *gcy‐21*, *F47D12.9* or *W02B12.12.* In addition, *glp‐1(−)* mutants downregulated fatty acid degradation, drug metabolism, autophagy, mitophagy, longevity regulating pathway, phagosome, which were also reversed by the RNAi *daf‐2*, *gcy‐21*, *F47D12.9* or *W02B12.12.* Generally, our data indicated that these dauer‐related genes rewired similar metabolism pattern in *glp‐1(−)* mutants.

### 2.6 NPR1/*gcy‐21* and TSSK6/*W02B12.12* were unfavourable prognostic indicators in glioma patients

**FIGURE 9:**
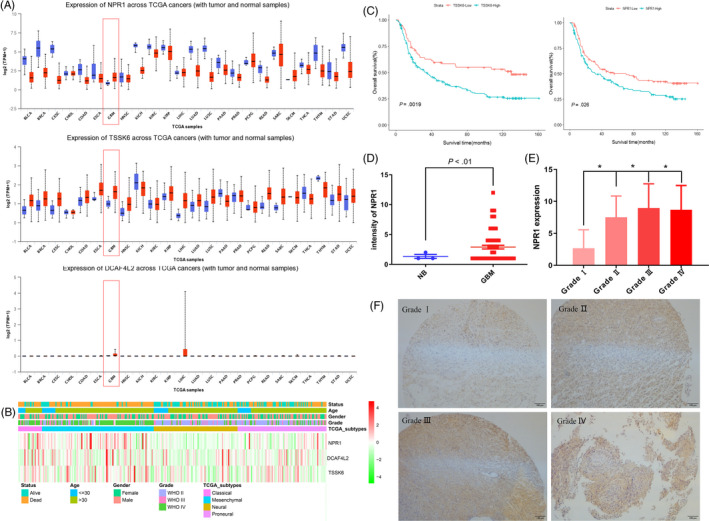
NPR1, DCAF4L2 and TSSK6 expression is increased in glioma, and elevated NPR1 or TSSK6 expression is a prognostic indicator of poor survival in patients with classical glioma. A, NPR1, TSSK6 and DCAF4L2 mRNA expression in multiple tumour tissues and normal tissues based on RNA‐Seq data from the TCGA. Log2‐normalized read count (TPM) is shown. TPM, transcripts per kilobase of exon model per million mapped reads. B, mRNA expression of NPR1, DCAF4L2 and TSSK6 was analysed in glioma tissues from the TCGA data sets. C, Kaplan–Meier survival curve analysis indicated that patients with NPR1 and TSSK6 overexpression had a significantly shorter overall survival in the classical subtype of glioma. D, E, NPR1 protein expression levels were elevated in primary glioma patient samples. F, Immunohistochemical staining of NPR1 in glioma tissue

*Gcy‐21*, *F47D12.9* and *W02B12.12* are homologous to NPR1, DCAF4L2 and TSSK6 in human, respectively.^40^ To translate our findings from *C elegans* models to human individuals, we first investigated whether the antitumour effect of these three genes was parallel in human. Using the TCGA data sets, we found that NPR1, DCAF4L2 and TSSK6 were overexpressed in glioma samples compared with adjacent normal tissues in human (Figure 9A). In Figure 9B, the cluster analysis revealed that NPR1, DCAF4L2 and TSSK6 were significantly upregulated in classical and mesenchymal subtypes of glioma. Next, we evaluated the prognostic values of NPR1, DCAF4L2 and TSSK6 in human glioma patients by Kaplan–Meier analysis. Patients with higher expression of NPR1 or TSSK6 suffered shorter overall survival (Figure 9C), while DCAF4L2 had no statistical significance (Figure S8). Furthermore, we detected the NPR1 protein expression levels in glioma tissues. Consistent with the above results, NPR1 was overexpressed in these patients and the expression level was positive correlated with tumour grade (Figure 9D‐F). Taken together, these results demonstrated that NPR1 and TSSK6 may be unfavourable prognostic factors and therapeutic targets for glioma patients.

## 3 DISCUSSION

The primary characteristic of tumour cells is highly proliferation and active energy metabolism.^6^ Previous studies indicated that reduced metabolism, such as calorie restriction, could inhibit tumorigenesis and progression.^12^, ^14^ Dormancy is a special process with extremely low metabolism in nature. Based on these two opposite metabolism states, we proposed that the activation of dormancy could suppress tumour progression. By using *C elegans*, we found that related genes significantly extended the lifespan of *glp‐1(−)* mutants via suppressing the germ cell proliferation and their human homolog genes had potential antitumour effect. This result suggested dauer gene screening in combination with tumour‐suppressing test in *glp‐1(−)* mutants provided a useful approach for antitumour target screening.

Given that protein kinases are a large family of enzymes and control many basic bioactivity including cell proliferation and cycling.^41^ Part of protein kinase has been considered to be oncogenic, as their transforming activity can determine the survival and proliferation of cancer cells.^42^, ^43^ Kinase‐specific inhibitors had high selectivity, efficiency and mild side effects than the cytotoxic drugs for chemotherapy.^44^, ^45^ Using this approach, the well‐known oncogenes (Tables 1 and 2), such as *akt‐2* and *let‐23,* could be recognized. *Akt‐2* encodes a homolog of the serine/threonine kinase AKT/PKB, drugs that target the phosphoinositide 3‐kinase (PI3K)/AKT pathway, which is frequently mutated in both solid tumours and haematologic malignancies.^46^, ^47^ *Let‐23*, an ortholog of human EGFR (epidermal growth factor receptor), is predicted to have epidermal growth factor‐activated receptor activity and is involved in several processes including determination of adult lifespan, male genitalia development and nematode larval development.^48^, ^49^, ^50^, ^51^ EGFR is a verified therapeutic target; antibody drugs such as cetuximab and panitumumab, which target the transmembrane protein epidermal growth factor receptor (EGFR), have made a major step forward in the treatment of cancer.^52^, ^53^

Intriguingly, our approach screened out three novel dauer‐related genes, namely *F47D12.9*, *W02B12.12* and *gcy‐21*, which could significantly extend the lifespan of *glp‐1(−)* mutants via suppressing tumour cell proliferation. The functions of these three genes in *C elegans* were not yet clear and these genes were conserved in human beings. We found that their homologous genes NPR1, DCAF4L2 and TSSK6 were highly expressed in glioma patients and patients with high NPR1 and TSSK6 predicted a short survival.

Studies reported that NPR1/*gcy‐21* is the receptor for the cardiac hormone atrial natriuretic peptide, and it has been reported to be highly expressed in cancer cells such as lung cancer, prostate cancer and ovarian cancer.^54^ The high expression and signalling of NPR1 are important for tumour growth; its deficiency protects C57BL/6 from lung, skin and ovarian cancers.^54^ Nojiri et al^55^ showed that atrial natriuretic peptide prevents cancer metastasis by suppressing the inflammatory reaction. DCAF4L2/*F47D12.9* is a member of WD‐repeat proteins. Wang et al^56^ found elevated DCAF4L2 expression in colorectal cancer was significantly correlated with clinical stage and it was an independent prognosis factor for survival. TSSK6/*W02B12.12* is a member of the serine/threonine protein kinase family, and it has been described as testis‐specific due to effects on fertility.^57^ Li et al^58^ discovered TSSK6 expression levels were positively correlated with T‐cell diversity in multiple cancers and TSSK6 could be a putative vaccine target in multiple cancer types. Overall, these three new genes are worth for further evaluation in tumour patients.

Current targets for tumour therapy could induce multiple side effects, indicating that targets with more safety are needed for treating cancers. There is no successful target therapy for glioma, and chemotherapy or radiotherapy is still the major strategy for glioma clinical treatment.^59^ P53 is a potential target to suppress tumour; however, P53 caused progeria in mouse.^60^ Our results suggested NPR1/*gcy‐21* and TSSK6/*W02B12.12* might be potential targets for glioma therapy via suppressing tumour cell proliferation (Figure 5). As an example, NPR1 interacts with atrial natriuretic peptide, which is an endogenous and physiological peptide, and atrial natriuretic peptide has significant antitumour effect in multiple cancers such as pancreatic cancer, breast cancer, small cell lung cancer, prostate cancer and colorectal cancer.^61^, ^62^

Notably, NPR1 and TSSK6 were highly correlated with the unfavourable prognosis of glioma patients and could be suitable prognostic indicators in glioma; these need to be confirmed by large clinical samples detection. NPR1 protein expression was increased in gastric, lung, skin, and ovarian tumours.^54^, ^63^ However, TCGA data sets showed that the mRNA expression of NPR1 has no significant difference in multiple cancers versus normal tissues. Generally, the mRNA expression level of genes was not always consistent with protein expression level, this non‐consistence of NPR1 expression at mRNA and protein level suggested that the regulation of NPR1 is complicated and the protein level modulations such as epigenetic regulation and post‐translation modifications possibly play important role in these tumours. In addition, the mRNA expression of TSSK6 is increased consistently in multiple tumours; however, its protein level in tumour tissues is unknown. Overall, few molecular biological works have been performed for the regulation of these two genes till now; the detailed mechanisms are under investigation. These findings just provide clues for glioma target therapy, which requires a lot of work to verify its function and reveal its mechanisms in human glioma cell and mouse.

In this study, we found that dauer‐related genes could extend the lifespan of *glp‐1(−)* mutants and this extension was independent of dauer stage and longevity (Figures 6 and 7), which suggests that these genes could directly suppress the tumour cell proliferation and could be potential targets for tumour clinical therapy. The profiles of mRNA expression showed that knockdown of *daf‐2*, *gcy‐21*, *F47D12.9* and *W02B12.12* shared similar characters of low metabolism in bioenergetics and biosynthetic needs compared to controls in *glp‐1(−)* mutants, and the rewired metabolism after the knockdown of *gcy‐21*/NPR1 and *W02B12.12*/TSSK6 possibly contributes to their antitumour effect. For example, we found knockdown of *daf‐2*, *gcy‐21*, *F47D12.9* and *W02B12.12* increased the fatty acid degradation in *glp‐1(−)* mutants. It has been reported that in cancer patients increased fatty acid degradation can suppress tumour development.^64^ Consistently, the level of fatty acid degradation was increased in low metabolic states, such as dormancy or fasting.^65^, ^66^ These results suggested that dauer‐related genes in *glp‐1(−)* mutants exert antitumour effects by rewiring tumour cell metabolism.

The experimental strengths and the similarities between the cellular and molecular processes present in *C elegans* and other animals across evolutionary time (metabolism, organelle structure and function, gene regulation, protein biology, etc) have made *C elegans* an excellent organism with which to study general metazoan biology.^67^, ^68^, ^69^ But no model organism can be used to answer all the research question, and working with *C elegans* has some limitations, for instance, *C elegans* lacks many genes that regulate cascades.^70^ *C elegans* as a good model for research in vivo, however, there are no *C elegans* cell lines exist. So our findings should be validated in higher eukaryotic model organisms, such as mammalian tumour cell lines and mice.

Conclusively, we found that dauer‐related gene extends the lifespan of *glp‐1(−)* mutants via suppressing the tumour cell proliferation and rewiring tumour cell metabolism. This work indicated that dauer gene screening in combination with tumour‐suppressing test in *glp‐1(−)* mutants provided a useful approach for antitumour target screening.

## 4 METHODS

### 4.1 Nematode strains and culture

We used the following strains in this study: wild‐type N2, CF1370 *daf‐2(e1370)*, CF1038 *daf‐16(mu86)*, GC833 *glp‐1(ar202)* were obtained from the Caenorhabditis Genetics Center. Worms are cultivated on standard NGM plates with *Escherichia coli* OP50. Nematodes were cultured at 20°C, except as noted below for temperature‐sensitive.

### 4.2 Worm RNAi by feeding

RNAi was performed essentially as per.^71^ Single colonies of HT115 bacteria containing *L4440* plasmids with cloned fragments corresponding to target genes were from RNAi feeding libraries. Each RNAi reagent was verified by DNA sequencing except for screening. Young adult hermaphrodites were placed onto NGM plates seeded with dsRNA‐expressing or empty vector control bacteria (RNAi feeding plate). After overnight incubation, worms were transferred to an identical fresh RNAi feeding plate and allowed to lay eggs for 2 hours.

### 4.3 Lifespan analysis

Lifespan was measured following previous protocols.^36^ For lifespan assays, we picked about 90 animals as L4 larvae at *t* = 0, and we transferred worms to OP50‐seeded or RNAi feeding plates (roughly 30 animals per plate) approximately every other day until the end of the reproductive period. Dead worms were counted every other day until all worms were dead. Worms that were missing or that died from extruded internal organs or from internally hatched progeny were censored and statistically incorporated into the lifespan analyses. All lifespan experiments were performed at 25°C. We generated lifespan curves using GraphPad Prism 7, and we determined *P* values using the Mantel‐Cox log‐rank test. Each lifespan experiment was repeated at least three times.

### 4.4 Fluorescence microscopy

For DAPI nuclear staining, gonads were dissected, fixed and stained with DAPI as described.^36^, ^72^ Intact worms were fixed in cold (−20°C) methanol for 5 minutes. Fixed worms were washed twice in modified M9 buffer (M9 without Mg2+), incubated 30 minutes in 100 ng/mL DAPI in modified M9 and washed two to three times in modified M9. To prepare dissected gonad preparations, animals of the desired age were picked onto a fresh plate containing no bacteria, immersed in 2 mL of phosphate‐buffered saline (PBS) containing 0.25 mM levamisole and transferred to a circular glass dish (3 cm diam and 1.5 cm deep). Worms were decapitated by slicing with two 25‐gauge syringe needles in the head region, which results in gonad extrusion. The preparations were fixed in 3 mL of 3% formaldehyde, 0.1 M K2HP04 (pH 7.2) for 2 hours Using a capillary pipette, worms with attached extruded gonads were transferred onto a 2% agarose pad covering most of a glass slide. After removing excess liquid with a capillary, extruded gonads were manipulated for optimal positioning with a drawn capillary and then overlaid with a 25 × 50‐mm coverslip. Z‐stack images were acquired for the same region in entire gonad, with a 20× water objective at 2‐μm intervals using a Leica Confocal Microscope. The germ cells in at least three worms for each group were detected and summarized for three independent trials. To quantify *C elegans* germ cell nuclear numbers, each entire z‐stack was loaded into FUJI as a single lei file. Then, we selected the Z‐stack image demonstrating the DAPI‐stained germ cells in the entire gonad arm for germ cell number analysis. If the two gonads were uneven size, germ cells from both gonads were measured and averaged. The average number was used to compare between groups. To distinguish undifferentiated germ cells from oocytes, only the undifferentiated proliferation germ cells, which exit pachytene, presenting nuclei fully enclosed by plasma membrane, have been detected.^73^

### 4.5 Quantitative RT‐PCR

More than 400 well‐fed synchronized worms on day 1 were collected into 1.5‐mL tube and washed at least three times with M9 buffer. Total RNA was extracted with Trizol reagent (Invitrogen).1 μg of total RNA was reverse‐transcribed in 20 μL using the PrimeScript™ RT reagent Kit with gDNA Eraser (TaKaRa). Real‐time RT‐PCR was carried out using the 7300 Real Time PCR System (Applied Biosystems) using PowerUp™ SYBR™ Green Master Mix (Applied Biosystems™). Expression level of each sample was standardized to *C elegans* actin endogenous control standard.^39^, ^41^ The following specific primers used were 5′‐TCTTCGTCGCATCACAACCATCAC‐3′ (forward) and 5′ GATCGCCTTGACTGCTACAACTCG‐3′ (reverse) for *W02B12.12*, product size was 195 bp, 5′‐CTCGTGTGGACGCTGATGTTACC‐3′ (forward) and 5′‐TGGTGAGTTGAGTGTTGGCATAGC‐3′ (reverse) for *F47D12.9*, product size was 152 bp, 5′‐CAGTGGCTGGAGGCTTCAAGTG‐3′ (forward) and 5′‐AGATGTTGGCACCGATGTTCAGAG‐3′ (reverse) for *gcy‐21*, product size was 136 bp, 5′‐CCCAATCCAAGAGAGGTATCCTT‐3′ (forward) and 5′ AGGTGTGATGCCAGATCTTCTCCA‐3′ (reverse) for *act‐1* as control, and product size was 120 bp. All these genes were annealed at 60°C. The folds of changes were shown as means ± SD. in three independent experiments with each triplicate.

### 4.6 Library preparation for Transcriptome sequencing

A total amount of 3 μg RNA per sample was used as input material for the RNA sample preparations. Sequencing libraries were generated using NEBNext^®^ UltraTM RNA Library Prep Kit for Illumina^®^ (NEB) following the manufacturer's recommendations and index codes were added to attribute sequences to each sample. PCR products were purified (AMPure XP system) and library quality was assessed on the Agilent Bioanalyzer 2100 system.

The clustering of the index‐coded samples was performed on a cBot Cluster Generation System using TruSeq PE Cluster Kit v3‐cBot‐HS (Illumia) according to the manufacturer's instructions. After cluster generation, the library preparations were sequenced on an Illumina Hiseq platform and 125/150 bp paired‐end reads were generated.

### 4.7 Data filtering

Raw data (raw reads) of fastq format were firstly processed through in‐house perl scripts. The raw data obtained by sequencing contain a small number of reads with sequencing linker or low quality sequencing. In order to ensure the quality and reliability of data analysis, the original data need to be filtered. The content of the filtering is as follows. Remove low quality reads (Qphred ≤ 20 the number of bases in the read length face = 50% above reads). In this step, clean data (clean reads) were obtained by removing reads containing adapter, reads containing ploy‐N and low quality reads from raw data. At the same time, the contents of Q20, Q30 and GC in the clean data were calculated. All the downstream analyses were based on the clean data with high quality.

### 4.8 Data analysis

Reference genome and gene model annotation files were downloaded from genome website directly (ftp://ftp.ensembl.org/pub/release‐88/fasta/caenorhabditis_elegans/). Index of the reference genome was built using Hisat2 v2.0.5, and paired‐end clean reads were aligned to the reference genome using Hisat2 v2.0.5.

Differential expression analysis of two conditions/groups (two biological replicates per condition) was performed using the DESeq2 R package (1.16.1). DESeq2 provides statistical routines for determining differential expression in digital gene expression data using a model based on the negative binomial distribution. The resulting *P*‐values were adjusted using the Benjamini and Hochberg's approach for controlling the false discovery rate. Genes with an adjusted *P*‐value < .05 found by DESeq2 were assigned as differentially expressed.

### 4.9 GO and KEGG enrichment analysis of differentially expressed genes

Gene Ontology (GO) enrichment analysis of differentially expressed genes was implemented by the clusterProfiler R package, in which gene length bias was corrected. GO terms with corrected *P* value <.05 were considered significantly enriched by differential expressed genes.

KEGG is a database resource for understanding high‐level functions and utilities of the biological system, such as the cell, the organism and the ecosystem, from molecular‐level information, especially large‐scale molecular datasets generated by genome sequencing and other high‐through put experimental technologies (http://www.genome.jp/kegg/). We used clusterProfiler R package to test the statistical enrichment of differential expression genes in KEGG pathways.

### 4.10 Sample collection

We included all available tumour samples from TCGA (https://tcgadata.nci.nih.gov). In addition, 77 glioma tumour samples and three non‐neoplastic normal brain tissues were obtained from Fudan University Minhang Hospital. All the samples were histologically graded according to the 2007 WHO Classification of Nervous System Tumors. The protocol was approved by the ethics committee of Minhang Hospital, and informed consent was signed by all participants.

### 4.11 Immunohistochemistry

The immunohistochemical analysis was performed on the 4‐mm‐thick fraction mounted on slides and sectioned from each tumour sample. Then, each slide was deparaffinized in 60°C, followed by treatment with xylene and graded alcohol. After the antigen retrieval and being blocked with 5% bovine serum albumin, tissue slides were incubated with antibodies overnight at 4°C against NPR1 (1:50, Invitrogen, PA5‐29049), DCAF4L2 (1:100, Proteintech, 21571‐1‐AP) and TSSK6 (1:50, H00083983‐M02) respectively, followed by the addition of the appropriate biotinylated secondary antibody for 30 minutes at room temperature. Each specimen was assigned a score according to the intensity of the staining (no staining = 0; weak staining = 1; moderate staining = 2; strong staining = 3) and the extent of stained cells (0% = 0, 1‐24% = 1, 25‐49% = 2, 50‐74% = 3, 75‐100% = 4). The final immunoreactive score was determined by multiplying the intensity score with the extent of score of stained cells, ranging from 0 (the minimum score) to 12 (the maximum score).

## CONFLICT OF INTEREST

No conflict of interest was declared.

## AUTHOR CONTRIBUTION

MYQ, HSF, ZSL and WLS designed the experiments; MYQ, HSF, ZSL, ZZY, KCY, CHL, LZM, CPR and HB performed experiments and statistical analysis; MYQ and ZSL wrote the manuscript; MYQ, ZSL and WLS revised manuscript. MYQ, HB and WLS provided funding support.

## Supporting information

Table S1‐S6

Figure S1‐S8

Figure S1‐S8_caption

## Data Availability

The authors declare that all the data supporting the findings of this study are available within the article and its Supplementary Information files and from the corresponding authors on reasonable request.
