# Supplementary material for: An approach using Caenorhabditis elegans screening novel targets to suppress tumour cell proliferation
Source: Cell Prolif. 2020 May 25;53(6):e12832. doi: 10.1111/cpr.12832 (PMC7309951; doi:10.1111/cpr.12832)
Supplement: Supplementary file 1 — Table S1‐S6 [file CPR-53-e12832-s001.docx]

Supplemental Table 1. Effect of classical dauer formation signals on lifespan

| 1. |  |  |  |  |
| --- | --- | --- | --- | --- |
| Genotype | T | Mean Lifespan | n | P value |
| *glp-1(+) L4440* RNAi | 25℃ | 10 | 68 |  |
| *glp-1(-) L4440* RNAi | 25℃ | 8 | 118 |  |
| *glp-1(-) daf-2* RNAi | 25℃ | 16 | 77 | <0.0001 |
| *glp-1(-)* *daf-1* RNAi | 25℃ | 10 | 141 | <0.0001 |
| *glp-1(-)* *daf-14* RNAi | 25℃ | 9 | 116 | <0.0001 |
| *glp-1(-)* *daf-11* RNAi | 25℃ | 9 | 117 | <0.0001 |
| *glp-1(-)* *tax-2* RNAi | 25℃ | 8 | 121 | <0.0001 |
| 2. |  |  |  |  |
| Genotype | T | Mean Lifespan | n | P value |
| *glp-1(+) L4440* RNAi | 25℃ | 10 | 91 |  |
| *glp-1(-) L4440* RNAi | 25℃ | 7 | 115 |  |
| *glp-1(-) daf-2* RNAi | 25℃ | 14.5 | 60 | <0.0001 |
| *glp-1(-)* *daf-1* RNAi | 25℃ | 9 | 111 | <0.0001 |
| *glp-1(-)* *daf-14* RNAi | 25℃ | 9 | 118 | <0.0001 |
| *glp-1(-)* *daf-11* RNAi | 25℃ | 10 | 136 | <0.0001 |
| *glp-1(-)* *tax-2* RNAi | 25℃ | 10 | 116 | <0.0001 |
| 3. |  |  |  |  |
| Genotype | T | Mean Lifespan | n | P value |
| *glp-1(+) L4440* RNAi | 25℃ | 12 | 86 |  |
| *glp-1(-) L4440* RNAi | 25℃ | 11 | 116 |  |
| *glp-1(-) daf-2* RNAi | 25℃ | 24 | 99 | <0.0001 |
| *glp-1(-)* *daf-1* RNAi | 25℃ | 13 | 141 | <0.0001 |
| *glp-1(-)* *daf-14* RNAi | 25℃ | 13 | 144 | <0.0001 |
| *glp-1(-)* *daf-11* RNAi | 25℃ | 13 | 134 | <0.0001 |
| *glp-1(-)* *tax-2* RNAi | 25℃ | 13 | 131 | <0.0001 |

*P-value of mean lifespan calculated by Log-rank (Mantel-Cox) Test.

Supplemental Table 2. Classical dauer formation signals extend the lifespan of *glp-1(-)* mutants via reducing the germ-line cell number

| Genotype/treatment | Estimated germ nuclei in most visible stack ± s.e | n | Reduction of  germ cell nuclei compared to *glp-1*(-) | P value |
| --- | --- | --- | --- | --- |
| day 4 adulthood |  |  |  |  |
| *glp-1(+)*  *L4440* RNAi | 364±60  (354±46)  (439±74) | 19  (11)  (10) |  |  |
| *glp-1(-)*  *L4440* RNAi | 777±118  (820±108)  (1015±130) | 16  (7)  (10) |  |  |
| *glp-1(-)*  *daf-2* RNAi | 436±108  (466±123)  (666±161) | 23  (12)  (14) | ~40%  (~40%)  (~30%) | <0.01  (<0.01)  (<0.01) |
| *glp-1(-)*  *daf-1* RNAi | 527±153  (448±95)  (782±175) | 21  (10)  (14) | ~30%  (~40%)  (~20%) | <0.01  (<0.01)  (<0.01) |
| *glp-1(-)*  *daf-14* RNAi | 487±136  (417±126)  (809±101) | 22  (9)  (14) | ~40%  (~50%)  (~20%) | <0.01  (<0.01)  (<0.01) |
| *glp-1(-)*  *daf-11* RNAi | 543±144  (530±157)  (688±152) | 23  (12)  (11) | ~30%  (~30%)  (~30%) | <0.01  (<0.01)  (<0.01) |
| *glp-1(-)*  *tax-2* RNAi | 616±99  (616±101)  (683±178) | 22  (11)  (14) | ~20%  (~25%)  (~30%) | <0.01  (<0.01)  (<0.01) |

*Additional repeats for the number of germ-line cell detected in each test are listed in parentheses.

*All P-values compare the genotype of corresponding row to that of the *L4440* RNAi row in *glp-1(-)* mutants.

Supplemental Table 3. Effect of new dauer related genes on lifespan in *glp-1(-)* mutants

| 1. |  |  |  |  |
| --- | --- | --- | --- | --- |
| Genotype | T | Mean Lifespan | n | P value |
| *glp-1(-) L4440* RNAi | 25℃ | 7 | 115 |  |
| *glp-1(-)* *gcy-21* RNAi | 25℃ | 10 | 90 | <0.0001 |
| *glp-1(-)* *F47D12.9* RNAi | 25℃ | 11 | 73 | <0.0001 |
| *glp-1(-)* *W02B12.12* RNAi | 25℃ | 10 | 83 | <0.0001 |
| 2. |  |  |  |  |
| Genotype | T | Mean Lifespan | n | P value |
| *glp-1(-) L4440* RNAi | 25℃ | 8 | 116 |  |
| *glp-1(-)* *gcy-21* RNAi | 25℃ | 10 | 121 | <0.0001 |
| *glp-1(-)* *F47D12.9* RNAi | 25℃ | 10 | 153 | <0.0001 |
| *glp-1(-)* *W02B12.12* RNAi | 25℃ | 10 | 145 | <0.0001 |
| 3. |  |  |  |  |
| Genotype | T | Mean Lifespan | n | P value |
| *glp-1(-) L4440* RNAi | 25℃ | 8 | 118 |  |
| *glp-1(-)* *gcy-21* RNAi | 25℃ | 9 | 108 | <0.01 |
| *glp-1(-)* *F47D12.9* RNAi | 25℃ | 11 | 111 | <0.0001 |
| *glp-1(-)* *W02B12.12* RNAi | 25℃ | 10 | 119 | <0.0001 |

*P-value of mean lifespan calculated by Log-rank (Mantel-Cox) Test.

Supplemental Table 4. Dauer related genes extend the lifespan of *glp-1(-)* mutants via reducing the germ-line cell number

| Genotype/  treatment | Estimated germ nuclei in most visible stack ± s.e | n | Reduction of  germ cell nuclei compared to *glp-1*(-) | P value |
| --- | --- | --- | --- | --- |
| day 4 adulthood |  |  |  |  |
| *glp-1(-)*  *L4440* RNAi | 776±295  (900±141)  (1016±157) | 6  (4)  (10) |  |  |
| *glp-1(-)*  *gcy-21* RNAi | 590±126  (549±92)  (779±134) | 24  (9)  (10) | (~20%)  (~20%) | <0.01  (<0.01)  (<0.01) |
| *glp-1(-)*  *F47D12.9* RNAi | 619±242  (689±111)  (635±175) | 10  (16)  (14) | (~10%)  (~30%) | <0.01  (<0.01)  (<0.01) |
| *glp-1(-)* *W02B12.12* RNAi | 578±218  (591±142)  (773±101) | 11  (16)  (3) | (~20%)  (~20%) | <0.01  (<0.01)  (<0.01) |

* Additional repeats for the number of germ-line cell detected in each test are listed in parentheses.

* All P-values compare the genotype of corresponding row to that of the *L4440* RNAi row in *glp-1(-)* mutants.

Supplemental Table 5. Effect of new dauer related genes on lifespan in N2 worms

| 1. |  |  |  |  |
| --- | --- | --- | --- | --- |
| Genotype | T | Mean Lifespan | n | P value |
| *glp-1(+) L4440* RNAi | 25℃ | 13 | 83 |  |
| *glp-1(+)* *gcy-21* RNAi | 25℃ | 12.5 | 120 | <0.0001 |
| *glp-1(+)* *F47D12.9* RNAi | 25℃ | 12 | 80 | <0.0001 |
| *glp-1(+)* *W02B12.12* RNAi | 25℃ | 13 | 37 | <0.0001 |
| 2. |  |  |  |  |
| Genotype | T | Mean Lifespan | n | P value |
| *glp-1(+) L4440* RNAi | 25℃ | 10 | 68 |  |
| *glp-1(+)* *gcy-21* RNAi | 25℃ | 10 | 74 |  |
| *glp-1(+)* *F47D12.9* RNAi | 25℃ | 10.5 | 77 |  |
| *glp-1(+)* *W02B12.12* RNAi | 25℃ | 10 | 85 |  |
| 3. |  |  |  |  |
| Genotype | T | Mean Lifespan | n | P value |
| *glp-1(+) L4440* RNAi | 25℃ | 10 | 91 |  |
| *glp-1(+)* *gcy-21* RNAi | 25℃ | 11 | 106 |  |
| *glp-1(+)* *F47D12.9* RNAi | 25℃ | 10 | 90 |  |
| *glp-1(+)* *W02B12.12* RNAi | 25℃ | 10 | 115 |  |

*P-value of mean lifespan calculated by Log-rank (Mantel-Cox) Test.

Supplemental Table 6. Effect of new dauer related genes on lifespan in different development stages

| L1 RNAi |  |  |  |  |
| --- | --- | --- | --- | --- |
| Genotype | T | Mean Lifespan | n | P value |
| *glp-1(-) L4440* RNAi | 25℃ | 7  (10) | 106  (128) |  |
| *glp-1(-)* *gcy-21* RNAi | 25℃ | 8  (12) | 123  (137) | <0.01  (<0.01) |
| *glp-1(-)* *F47D12.9* RNAi | 25℃ | 9  (12) | 131  (115) | <0.01  (<0.01) |
| *glp-1(-)* *W02B12.12* RNAi | 25℃ | 9  (12) | 124  (130) | <0.01  (<0.01) |
| *glp-1(-)* *daf-2* RNAi | 25℃ | 11.5 | 64 | <0.01 |
|  |  |  |  |  |
| L2 RNAi |  |  |  |  |
| Genotype | T | Mean Lifespan | n | P value |
| *glp-1(-) L4440* RNAi | 25℃ | 6  (6) | 75  (86) |  |
| *glp-1(-)* *gcy-21* RNAi | 25℃ | 7  (8) | 100  (98) | <0.01  (<0.01) |
| *glp-1(-)* *F47D12.9* RNAi | 25℃ | 8  (8) | 87  (87) | <0.01  (<0.01) |
| *glp-1(-)* *W02B12.12* RNAi | 25℃ | 8  (8) | 121  (121) | <0.01  (<0.01) |
| *glp-1(-)* *daf-2* RNAi | 25℃ | 13 | 44 | <0.01 |
|  |  |  |  |  |
| L3 RNAi |  |  |  |  |
| Genotype | T | Mean Lifespan | n | P value |
| *glp-1(-) L4440* RNAi | 25℃ | 7  (7) | 120  (59) |  |
| *glp-1(-)* *gcy-21* RNAi | 25℃ | 9  (9) | 141  (71) | <0.01  (<0.01) |
| *glp-1(-)* *F47D12.9* RNAi | 25℃ | 9  (11) | 130  (113) | <0.01  (<0.01) |
| *glp-1(-)* *W02B12.12* RNAi | 25℃ | 9  (14) | 119  (92) | <0.01  (<0.01) |
| *glp-1(-)* *daf-2* RNAi | 25℃ | 13 | 66 | <0.01 |
|  |  |  |  |  |
| L4 RNAi |  |  |  |  |
| Genotype |  |  |  |  |
| *glp-1(-) L4440* RNAi | 25℃ | 7  (7) | 110  (77) |  |
| *glp-1(-)* *gcy-21* RNAi | 25℃ | 9  (9) | 115  (124) | <0.01  (<0.01) |
| *glp-1(-)* *F47D12.9* RNAi | 25℃ | 9  (9) | 128  (99) | <0.01  (<0.01) |
| *glp-1(-)* *W02B12.12* RNAi | 25℃ | 9  (11) | 124  (125) | <0.01  (<0.01) |
| *glp-1(-)* *daf-2* RNAi | 25℃ | 16 | 28 | <0.01 |
|  |  |  |  |  |
| Day 1 of adulthood RNAi |  |  |  |  |
| Genotype | 25℃ |  |  |  |
| *glp-1(-) L4440* RNAi | 25℃ | 6.5  (7) | 76  (76) |  |
| *glp-1(-)* *gcy-21* RNAi | 25℃ | 9  (11) | 81  (81) | <0.01  (<0.01) |
| *glp-1(-)* *F47D12.9* RNAi | 25℃ | 10  (11) | 78  (78) | <0.01  (<0.01) |
| *glp-1(-)* *W02B12.12* RNAi | 25℃ | 11  (11) | 78  (75) | <0.01  (<0.01) |
| *glp-1(-)* *daf-2* RNAi | 25℃ | 11 | 51 | <0.01 |
|  |  |  |  |  |
| Day 4 of adulthood RNAi |  |  |  |  |
| Genotype |  |  |  |  |
| *glp-1(-) L4440* RNAi | 25℃ | 7  (6.5) | 100  (86) |  |
| *glp-1(-)* *gcy-21* RNAi | 25℃ | 8  (9) | 102  (98) | <0.01  (<0.01) |
| *glp-1(-)* *F47D12.9* RNAi | 25℃ | 8  (8) | 107  (78) | <0.01  (<0.01) |
| *glp-1(-)* *W02B12.12* RNAi | 25℃ | 8  (9) | 107  (93) | <0.01  (<0.01) |
| *glp-1(-)* *daf-2* RNAi | 25℃ | 11 | 51 | <0.01 |

*P-value of mean lifespan calculated by Log-rank (Mantel-Cox) Test.
