# Supplementary material for: An approach using Caenorhabditis elegans screening novel targets to suppress tumour cell proliferation
Source: Cell Prolif. 2020 May 25;53(6):e12832. doi: 10.1111/cpr.12832 (PMC7309951; doi:10.1111/cpr.12832)
Supplement: Supplementary file 2 — Figure S1‐S8 [file CPR-53-e12832-s002.pptx]

## Slide 1
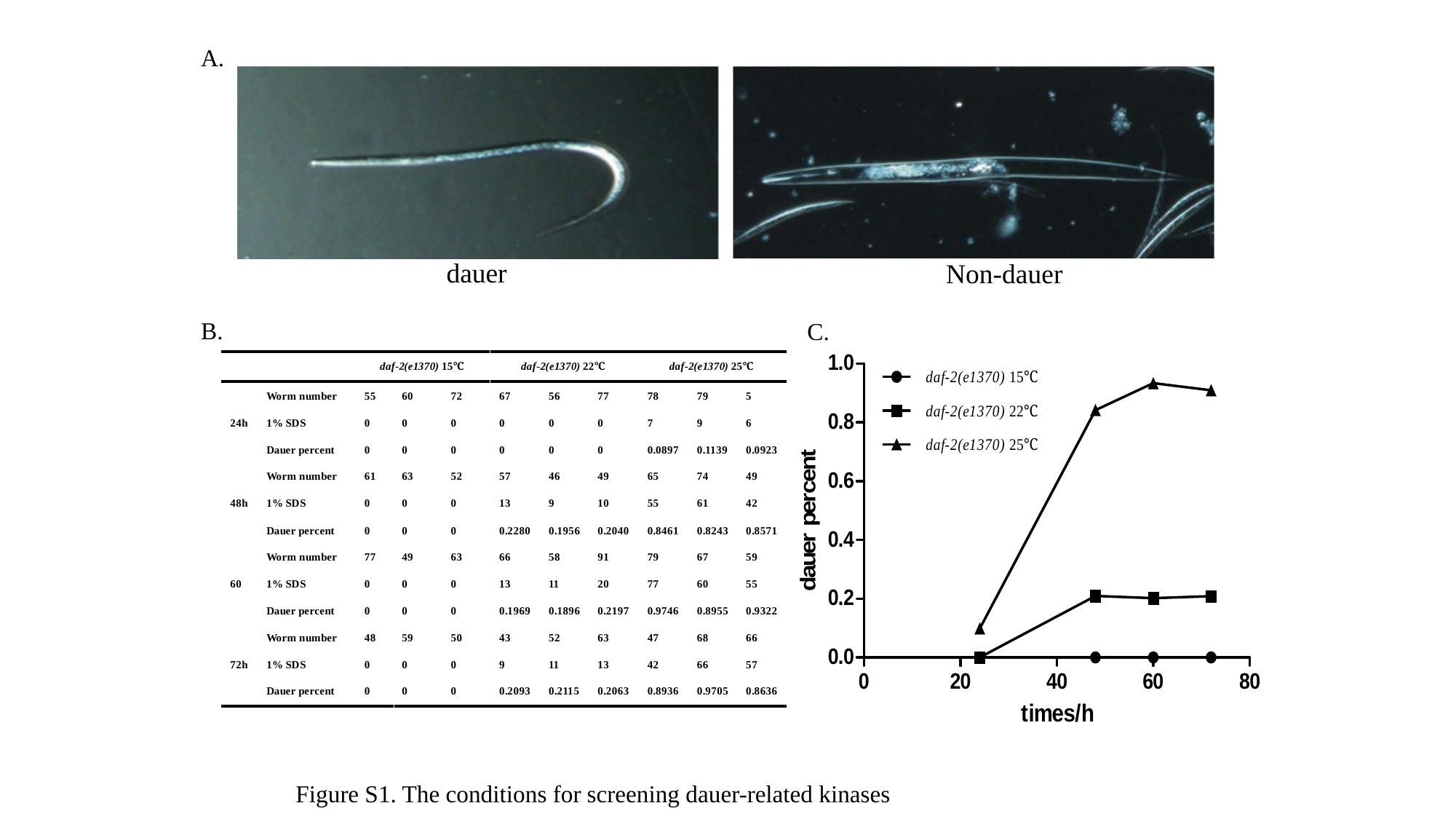

A.
dauer
Non-dauer
B.
C.
Figure S1. The conditions for screening dauer-related kinases

## Slide 2
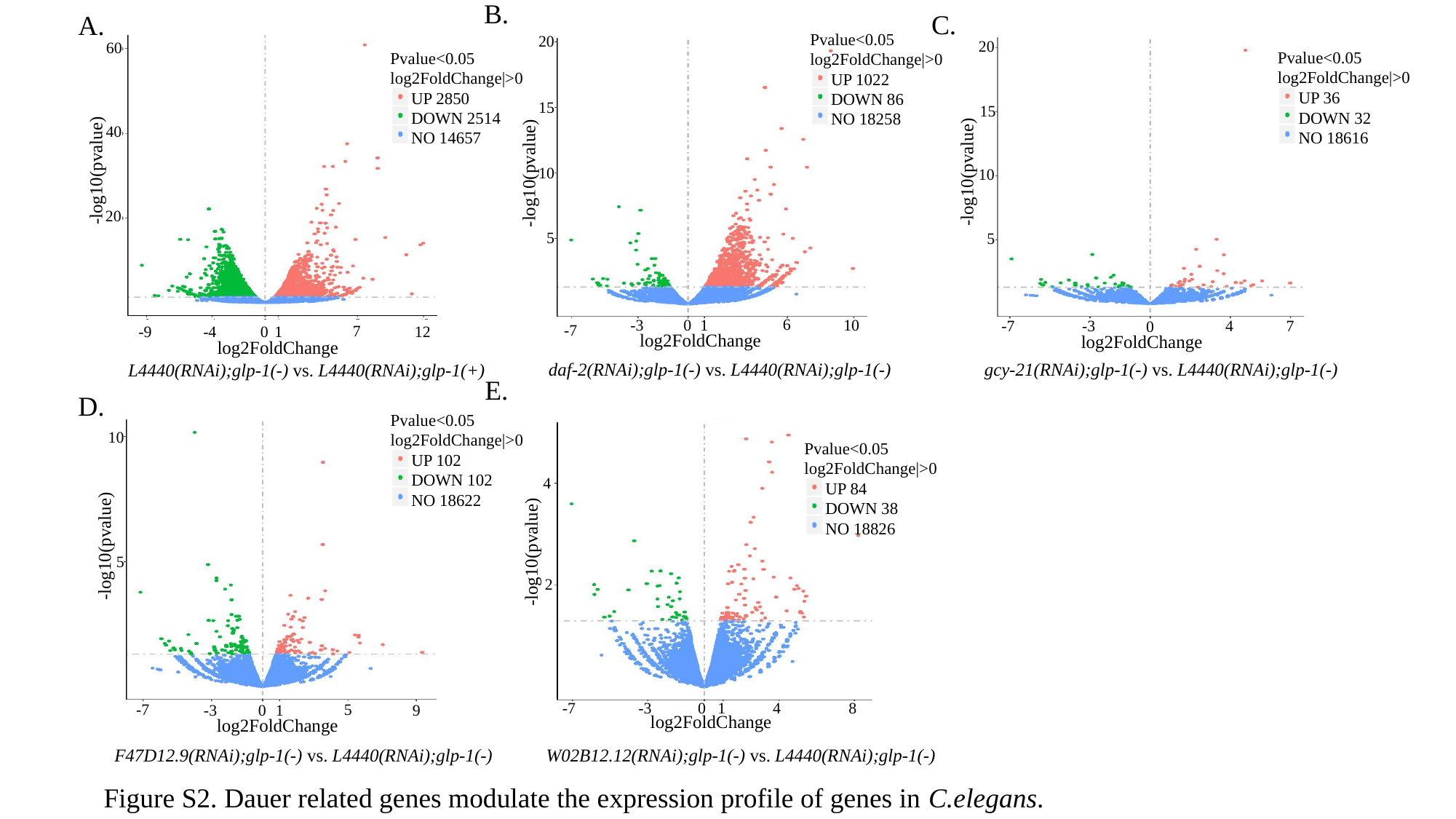

B.
C.
A.
Pvalue<0.05
log2FoldChange|>0
 UP 1022
 DOWN 86
 NO 18258
20
15
-log10(pvalue)
10
5
6
10
-3
1
0
-7
log2FoldChange
20
Pvalue<0.05
log2FoldChange|>0
 UP 36
 DOWN 32
 NO 18616
15
-log10(pvalue)
10
5
4
-7
7
-3
0
log2FoldChange
60
Pvalue<0.05
log2FoldChange|>0
 UP 2850
 DOWN 2514
 NO 14657
40
-log10(pvalue)
20
7
-9
12
-4
1
0
log2FoldChange
daf-2(RNAi);glp-1(-) vs. L4440(RNAi);glp-1(-)
gcy-21(RNAi);glp-1(-) vs. L4440(RNAi);glp-1(-)
L4440(RNAi);glp-1(-) vs. L4440(RNAi);glp-1(+)
E.
D.
Pvalue<0.05
log2FoldChange|>0
 UP 102
 DOWN 102
 NO 18622
10
-log10(pvalue)
5
5
-7
9
-3
1
0
log2FoldChange
Pvalue<0.05
log2FoldChange|>0
 UP 84
 DOWN 38
 NO 18826
4
-log10(pvalue)
2
8
0
4
-7
-3
1
log2FoldChange
F47D12.9(RNAi);glp-1(-) vs. L4440(RNAi);glp-1(-)
W02B12.12(RNAi);glp-1(-) vs. L4440(RNAi);glp-1(-)
Figure S2. Dauer related genes modulate the expression profile of genes in C.elegans.

## Slide 3
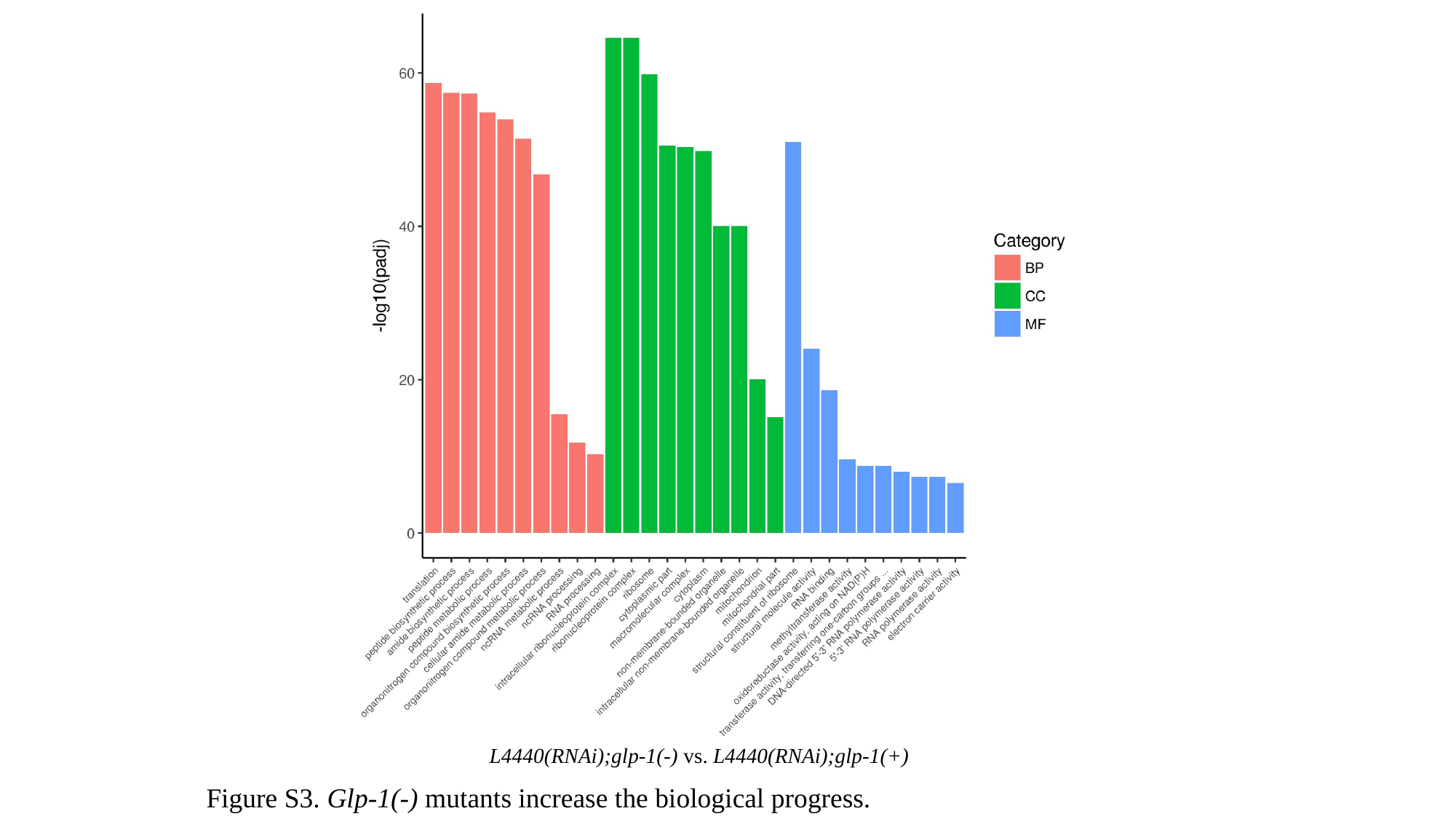

L4440(RNAi);glp-1(-) vs. L4440(RNAi);glp-1(+)
Figure S3. Glp-1(-) mutants increase the biological progress.

## Slide 4
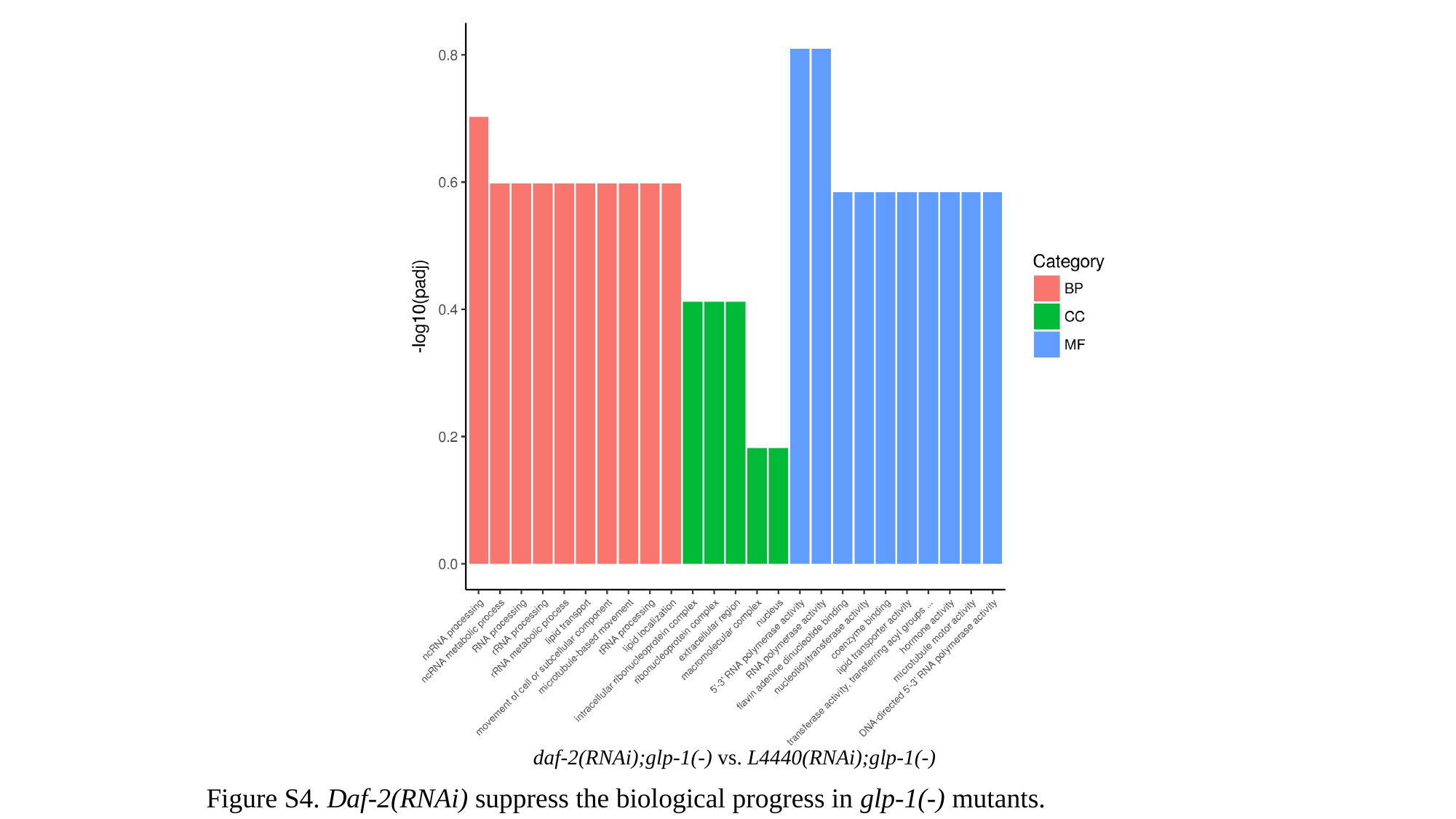

daf-2(RNAi);glp-1(-) vs. L4440(RNAi);glp-1(-)
Figure S4. Daf-2(RNAi) suppress the biological progress in glp-1(-) mutants.

## Slide 5
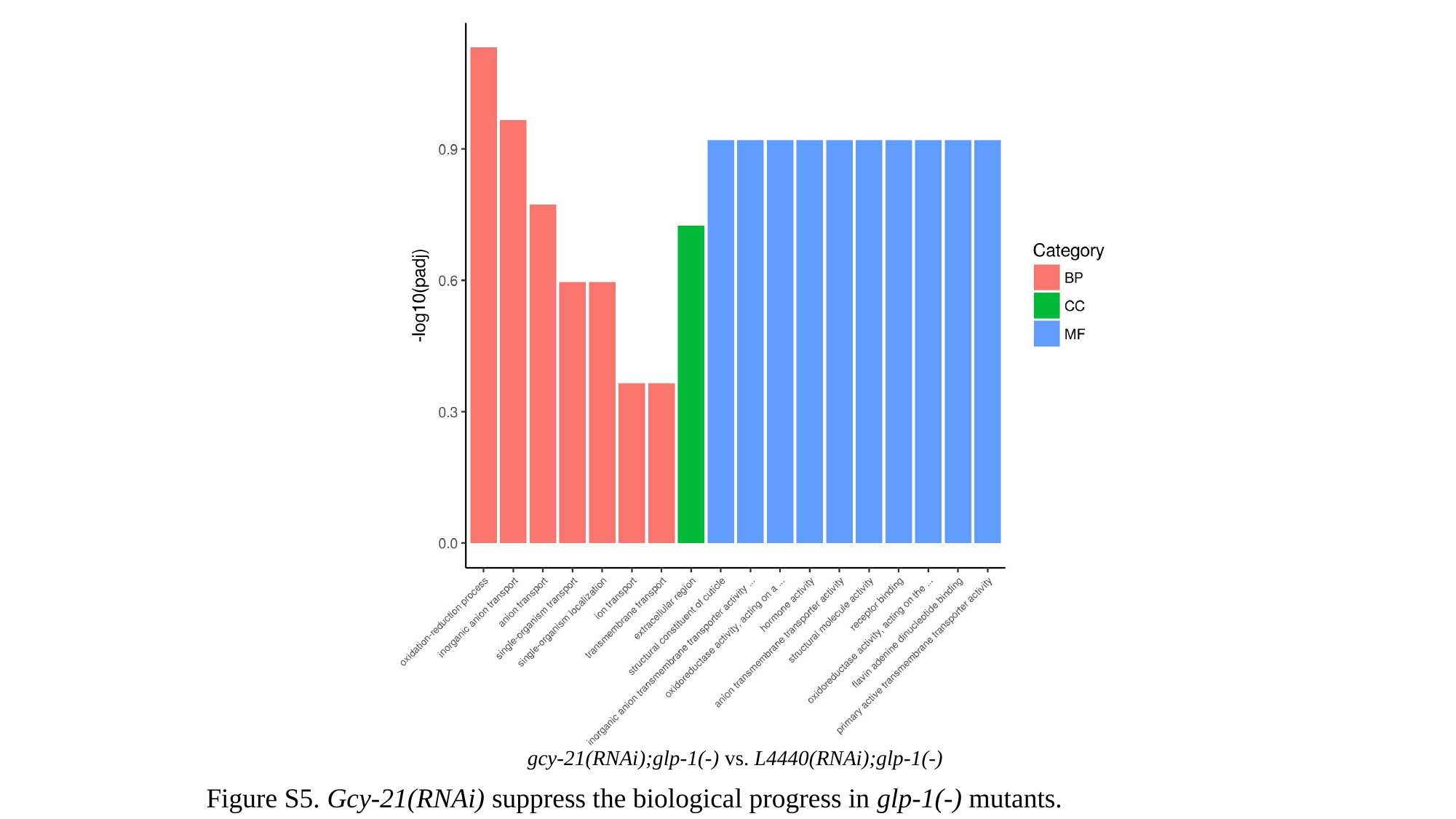

gcy-21(RNAi);glp-1(-) vs. L4440(RNAi);glp-1(-)
Figure S5. Gcy-21(RNAi) suppress the biological progress in glp-1(-) mutants.

## Slide 6
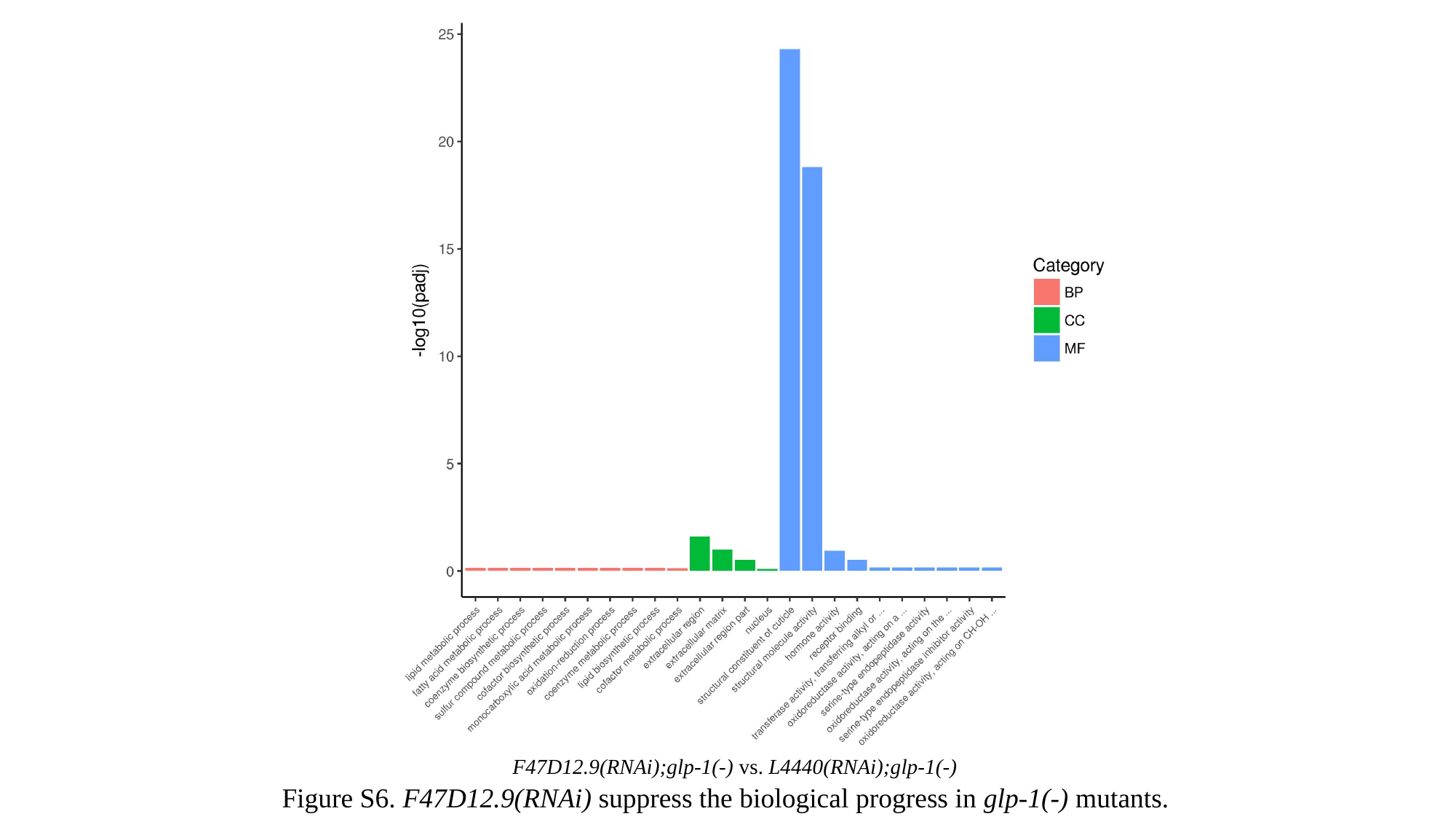

F47D12.9(RNAi);glp-1(-) vs. L4440(RNAi);glp-1(-)
Figure S6. F47D12.9(RNAi) suppress the biological progress in glp-1(-) mutants.

## Slide 7
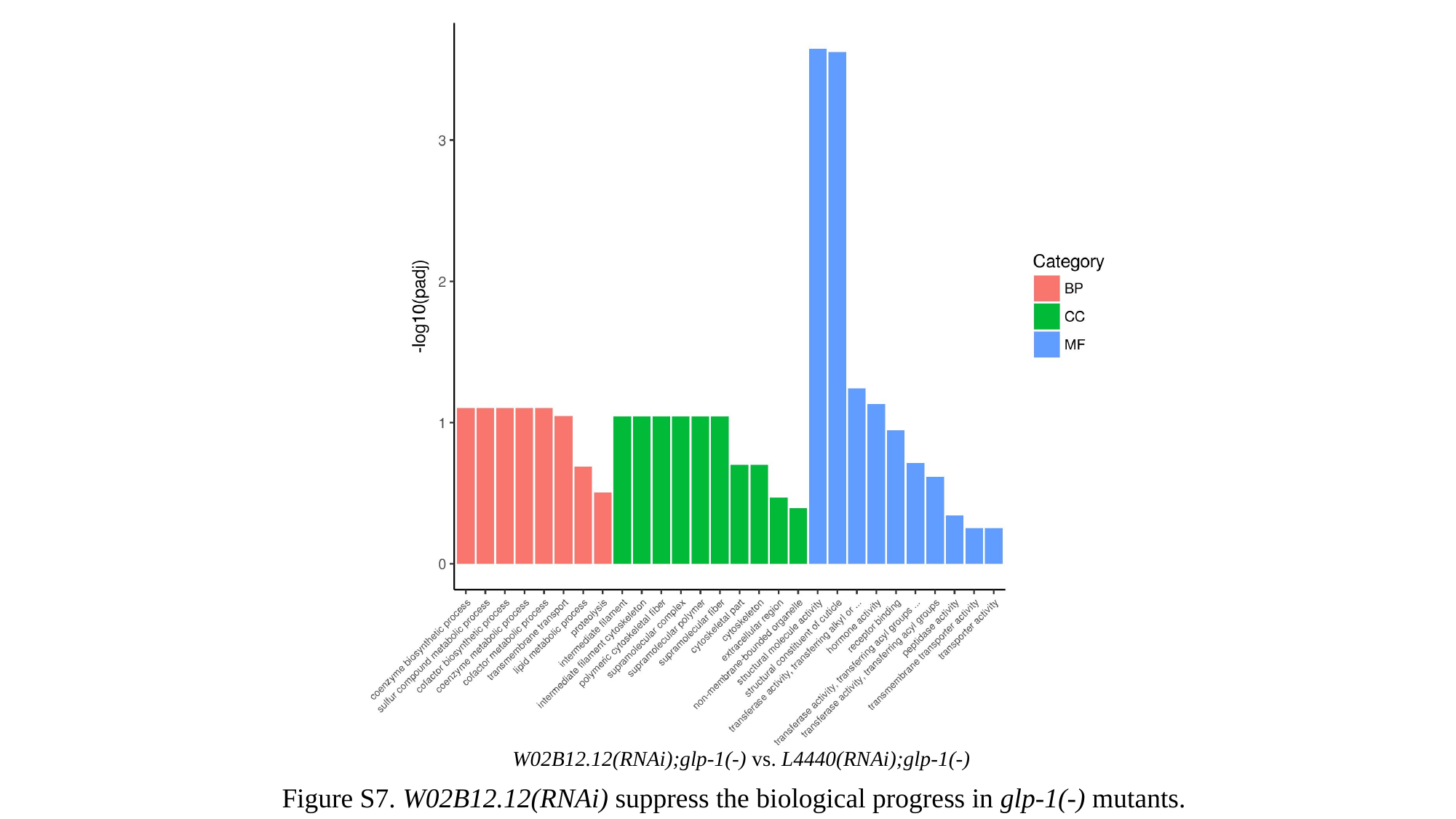

W02B12.12(RNAi);glp-1(-) vs. L4440(RNAi);glp-1(-)
Figure S7. W02B12.12(RNAi) suppress the biological progress in glp-1(-) mutants.

## Slide 8
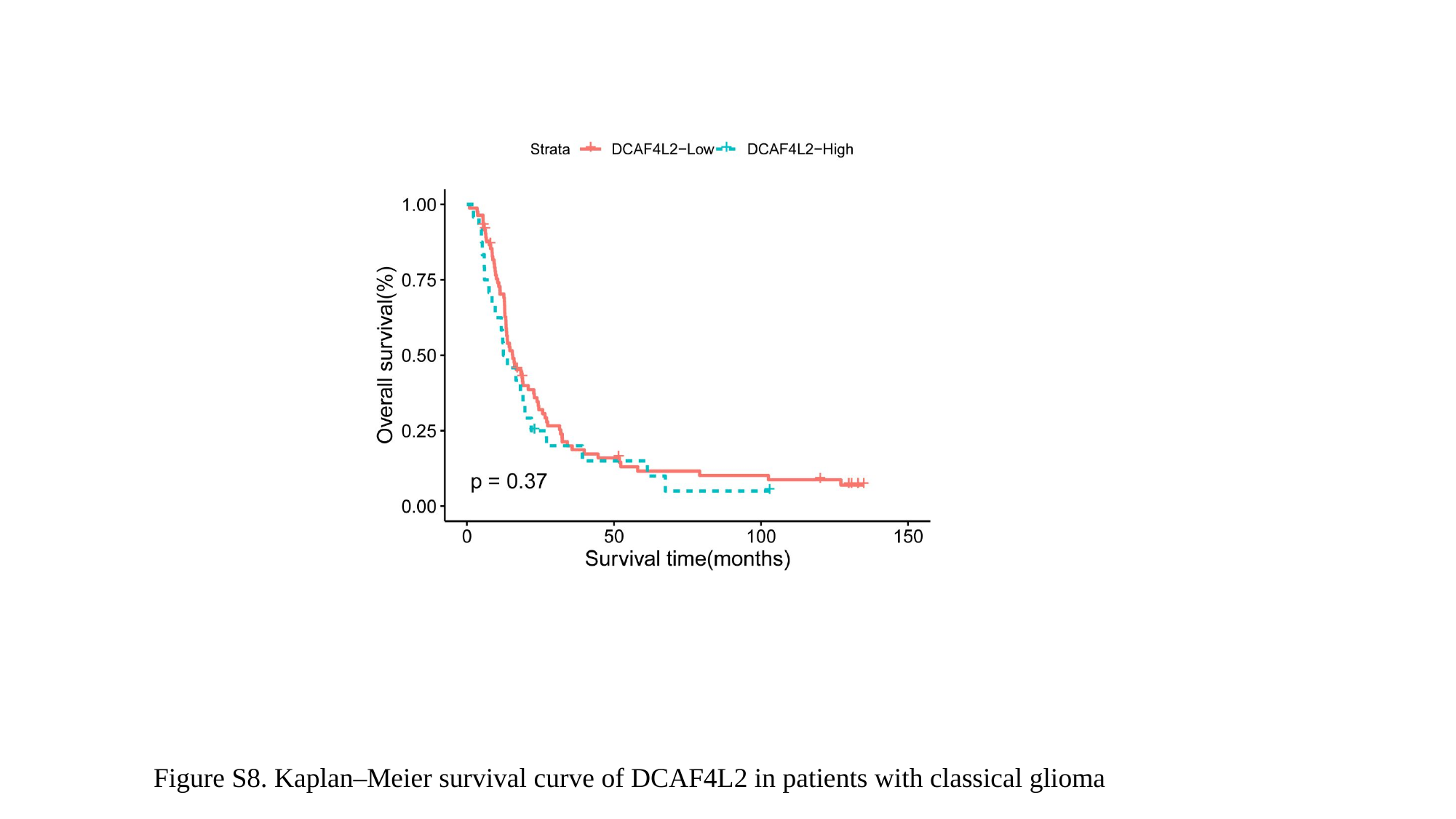

Figure S8. Kaplan–Meier survival curve of DCAF4L2 in patients with classical glioma
