# Supplementary material for: An approach using Caenorhabditis elegans screening novel targets to suppress tumour cell proliferation
Source: Cell Prolif. 2020 May 25;53(6):e12832. doi: 10.1111/cpr.12832 (PMC7309951; doi:10.1111/cpr.12832)
Supplement: Supplementary file 3 — Figure S1‐S8_caption [file CPR-53-e12832-s003.docx]

**Figure S1. The conditions for screening dauer-related kinases**

1. The morphology of dauer after 1% SDS treatment with *daf-2(e1370)*.

(B) The percentage of dauer formation at different temperature and time in the background of *daf-2(e1370)*.

(C) Relative dauer formation curves of worms.

**Figure S2. Dauer-related genes modulate the expression profile of genes in *glp-1(-)* mutants.**

Mechanism study of dauer-related genes extend the lifespan of *glp-1(-)* mutants. RNA was extract after RNAi treatment. (A) The differential genes of *L4440 (RNAi); glp-1(-)* compared with *L4440 (RNAi); glp-1(+)* mutants. (B) The differential genes of *daf-2* compared with *L4440* in the background of *glp-1(-)* mutants. (C) The differential genes of *gcy-21* compared with *L4440* in the background of *glp-1(-)* mutants. (D) The differential genes of *F47D12.9* compared with *L4440* in the background of *glp-1(-)* mutants. (E) The differential genes of *W02B12.12* compared with *L4440* in the background of *glp-1(-)* mutant. *Glp-1(+)* refers to N2 worms. Blue represented genes were not changed, red represented upregulated genes, green represented downregulated genes.

**Figure S3. *Glp-1(-)* mutants increase the biological progress.**

GO enrichment analysis of the identified differentially expressed genes. The 20 most significantly enriched categories are shown. (A) Gene Ontology analysis for the upregulated genes of *glp-1(+)* compared with *glp-1(-)* mutants. BP, biological; CC, cellular component; MF, molecular function.

**Figure S4. *Daf-2(RNAi)* suppress the biological progress in *glp-1(-)* mutants.**

GO enrichment analysis of the identified differentially expressed genes. The 20 most significantly enriched categories are shown. (A) Gene Ontology analysis for the upregulated genes of *daf-2(RNAi); glp-1(-)* compared with *L4440 (RNAi)*; *glp-1(-)*. BP, biological; CC, cellular component; MF, molecular function.

**Figure S5. *Gcy-21(RNAi)* suppress the biological progress in *glp-1(-)* mutants.**

GO enrichment analysis of the identified differentially expressed genes. The 20 most significantly enriched categories are shown. (A) Gene Ontology analysis for the upregulated genes of *gcy-21(RNAi); glp-1(-)* compared with *L4440 (RNAi)*; *glp-1(-)*. BP, biological; CC, cellular component; MF, molecular function.

**Figure S6. *F47D12.9 (RNAi)* suppress the biological progress in *glp-1(-)* mutants.**

GO enrichment analysis of the identified differentially expressed genes. The 20 most significantly enriched categories are shown. (A) Gene Ontology analysis for the upregulated genes of *F47D12.9 (RNAi); glp-1(-)* compared with *L4440 (RNAi)*; *glp-1(-)*. BP, biological; CC, cellular component; MF, molecular function.

**Figure S7. *W02B12.12 (RNAi)* suppress the biological progress in *glp-1(-)* mutants.**

GO enrichment analysis of the identified differentially expressed genes. The 20 most significantly enriched categories are shown. (A) Gene Ontology analysis for the upregulated genes of *W02B12.12 (RNAi); glp-1(-)* compared with *L4440 (RNAi)*; *glp-1(-)*. BP, biological; CC, cellular component; MF, molecular function.

**Figure S8. Kaplan–Meier survival curve of DCAF4L2 in patients with classical glioma**

A. Kaplan–Meier survival curve of DCAF4L2 in the classical subtype of glioma.
